# Supplementary material for: Exploring bat-inspired cyclic tryptophan diketopiperazines as ABCB1 Inhibitors
Source: Commun Chem. 2024 Jul 13;7:158. doi: 10.1038/s42004-024-01225-z (PMC11246513; doi:10.1038/s42004-024-01225-z)

Exploring bat-inspired cyclic tryptophan diketopiperazines as ABCB1 Inhibitors.

Supplementary items

Supplementary Fig. S1 Chemical structures.

Supplementary Fig. S2 Computational modelling of ABCB1-ligand binding.

Supplementary Fig. S3 Scheme of *Cyclo*-(L-Trp-L-Trp) benzylation.

Supplementary Fig. S4 Rhodamine 123 accumulation assay for *Cyclo*-(L-1-methyl-Trp-L-1-methyl-Trp) by flow cytometry.

Supplementary Fig. S5 Rhodamine 123 accumulation assay for C3N-Dbn-Trp2 by flow cytometry.

Supplementary Fig. S6 Rhodamine 123 accumulation assay for benzylated *Cyclo*-(L-Trp-L-Trp) compounds by flow cytometry.

Supplementary Fig. S7 C3N-Dbn-Trp2 inhibits ABCB1 efflux and sensitises cells to doxorubicin-induced cell death.

Supplementary Fig. S8 CRISPR *ABCB1* knockout of HCT-15 cells.

Supplementary Fig. S9 Images of Western blot membranes in Supplementary Fig. 1a and 2e.

Supplementary Fig. S10 Images of Western blot membranes in Fig. 3a and 3d.

Supplementary Fig. S11 Images of Western blot membranes in Fig. 8a.

Supplementary Fig. S12 Images of Western blot membranes in Fig. 8b, 8c, and 8f.

Supplementary Fig. S13 Images of Western blot membranes in Supplementary Fig. S8.

Supplementary Table S1 Molecular interactions of the compounds for truncated ABCB1 model during molecular dynamics simulations.

Supplementary Information for benzylation of *cyclo*-L-Trp-L-Trp-DKP

Supplementary Video 1: Molecular interactions of the compounds with ABCB1.

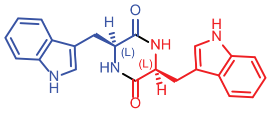

***Cyclo*-(L-Trp-L-Trp)**

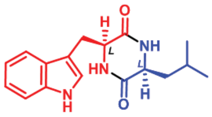

***Cyclo*-(L-Leu-L-Trp)**

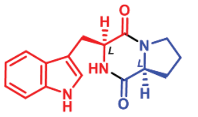

***Cyclo*-(L-Trp-L-Pro)**

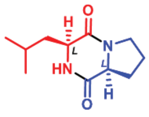

***Cyclo*-(L-Leu-L-Pro)**

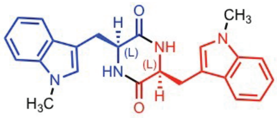

***Cyclo*-(L-1-methyl-Trp-L-1-methyl-Trp)**

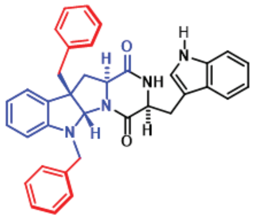

C3N-Dbn-Trp2

Supplementary Fig. S1 Chemical structures.

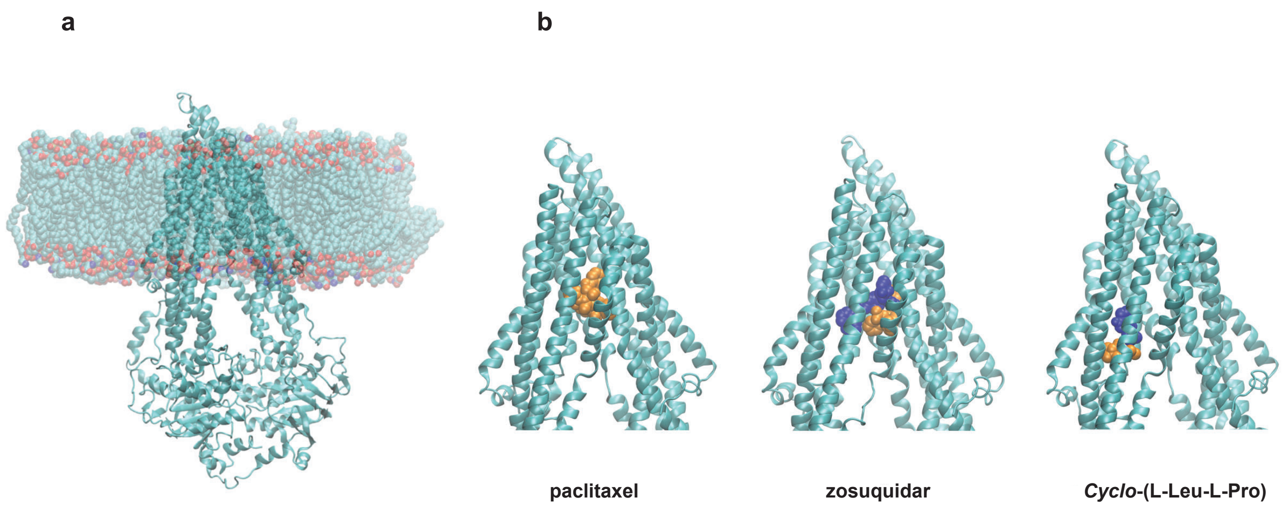

Supplementary Fig. S2 Computational modelling of ABCB1-ligand binding.

(a) Computational modelling of ABCB1. The system contains ABCB1 (pdb-ID:6QEX) embedded within a plasma membrane model, surrounded by a physiological salt solution.
(b) Predicted binding modes of paclitaxel, zosuquidar, and *Cyclo*-(L-Lue-L-Pro) to ABCB1. Blue or orange colour represents one molecule each. The top half of the ABCB1 structure is shown.

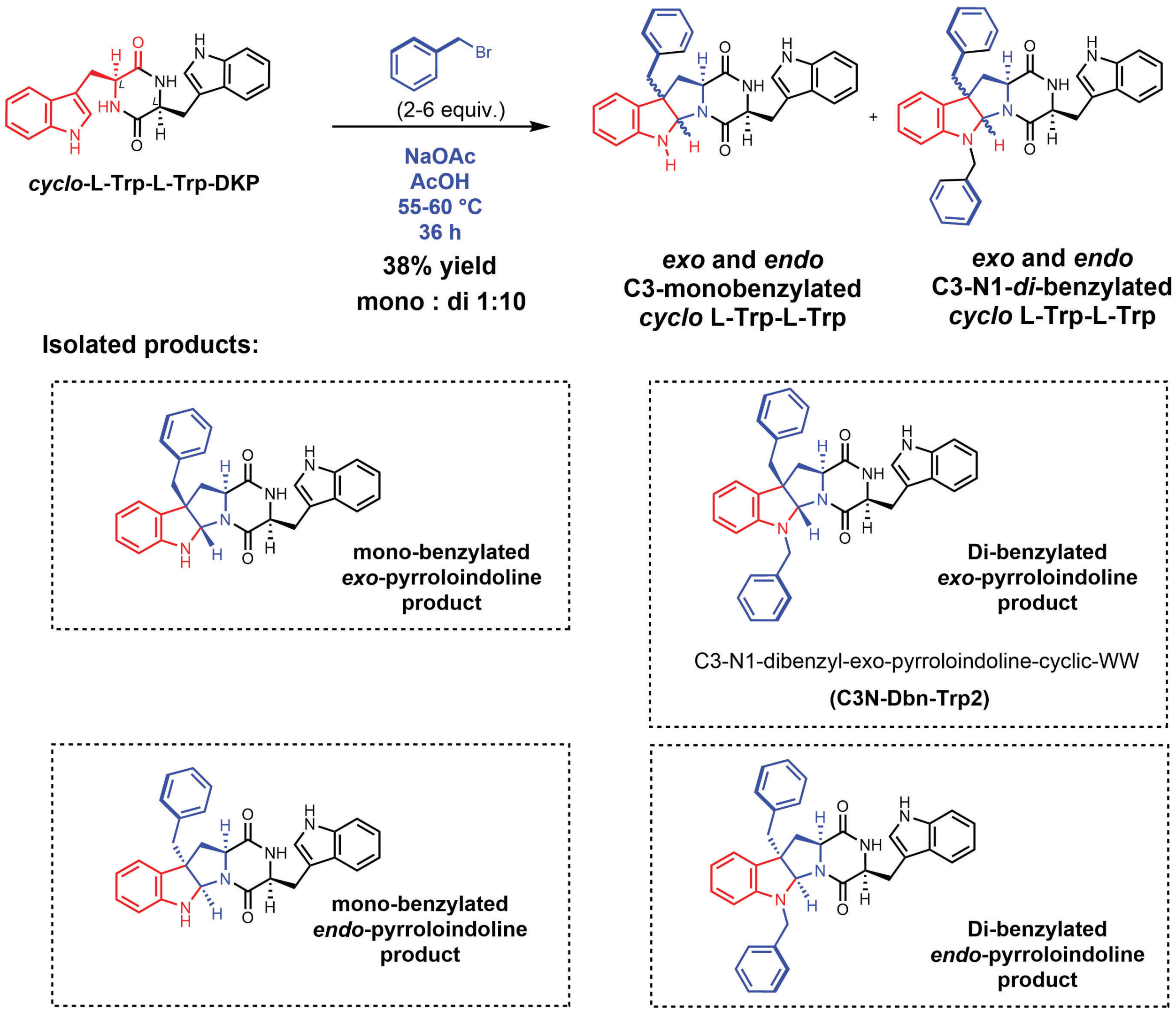

Supplementary Fig. S3 Scheme of Cyclo-(L-Trp-L-Trp) benzylation.

C3- and N1-benzylation of *Cyclo*-(L-Trp-L-Trp) results in the four derivatized products. C3N-Dbn-Trp2 was purified from a mixture of four products.

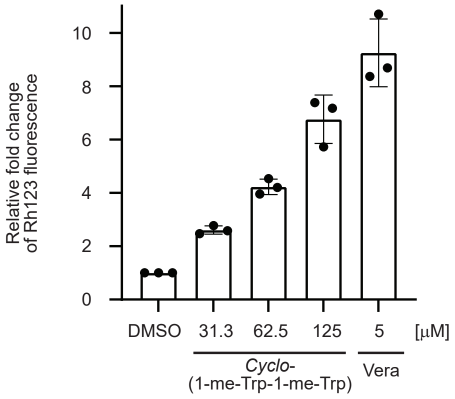

Supplementary Fig. S4 Rhodamine 123 accumulation assay for *Cyclo*-(L-1-methyl-Trp-L-1-methyl-Trp) by flow cytometry.

Parental PaKiT03 cells were pre-treated with the indicated amount of *Cyclo*-(L-1-methyl-Trp-L-1-methyl-Trp) or verapamil (Vera, 5 μM) before incubating with 2.5 μM Rh123. Bar graphs represent the mean (± SD of three independent experiments) fluorescent intensity of Rh123 relative to the DMSO-treated cells (the first bar).

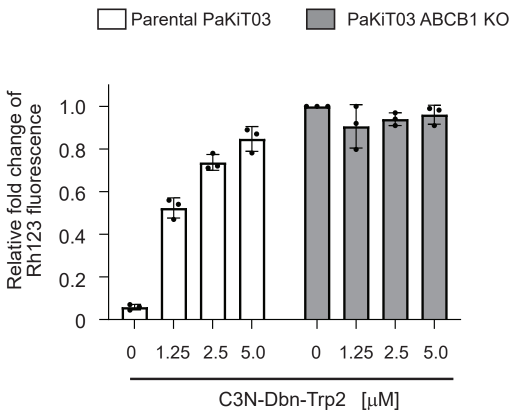

Supplementary Fig. S5 Rhodamine 123 accumulation assay for C3N-Dbn-Trp2 by flow cytometry.

Parental PaKiT03 and *ABCB1* KO cells were pre-treated with the indicated amount of C3N-Dbn-Trp2 before incubating with 2.5 μM Rh123. Bar graphs represent the mean (± SD of three independent experiments) fluorescent intensity of Rh123 relative to the DMSO-treated PaKiT03 *ABCB1* KO cells. White bars: parental PakiT03 cells; Gray bars: PakiT03 ABCB1 KO cells.

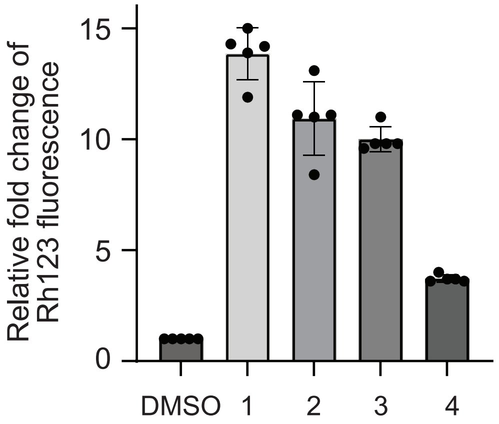

1

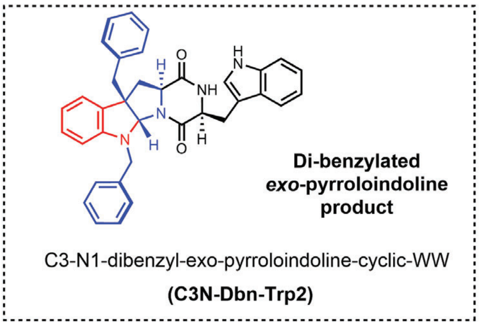

2

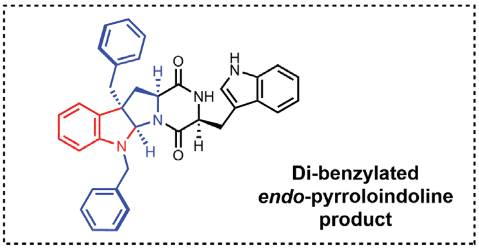

3

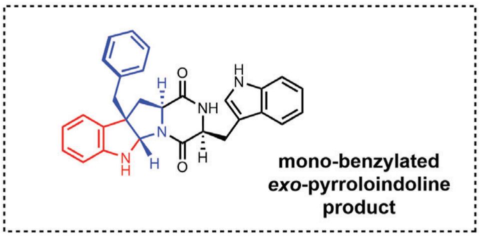

4

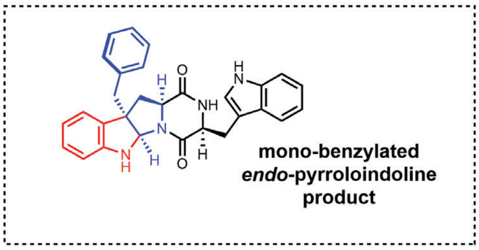

Supplementary Fig. S6 Rhodamine 123 accumulation assay for benzylated *Cyclo*-(L-Trp-L-Trp) compounds by flow cytometry.

PaKiT03 cells were pre-treated with 10 μM of the indicated compounds before incubating with 2.5 μM Rh123. Bar graphs represent the mean (± SD of five independent experiments) fluorescent intensity of Rh123 relative to the DMSO-treated PaKiT03 cells.

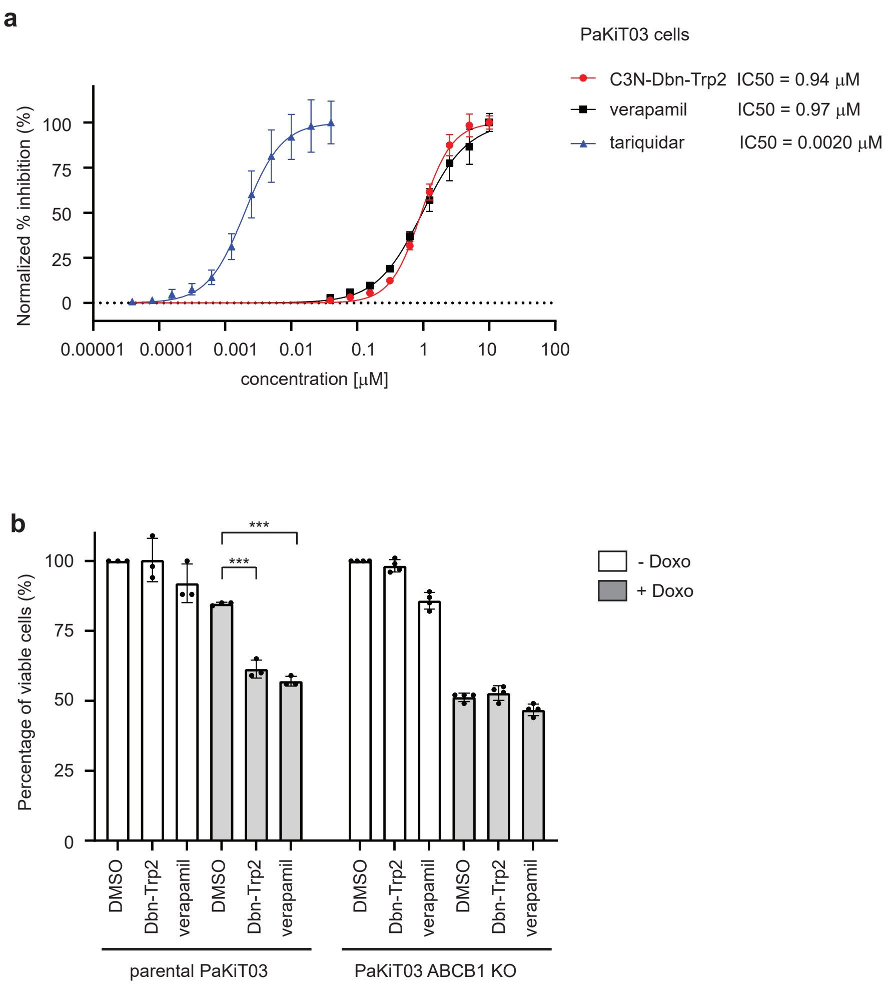

Supplementary Fig. S7 C3N-Dbn-Trp2 inhibits ABCB1 efflux and sensitises cells to doxorubicin-induced cell death.

(a) Flow cytometry analysis of rhodamine 123 (Rh123) accumulation. PaKiT03 parental cells were pre-treated with different concentrations of C3N-Dbn-Trp2, verapamil or tariquidar before incubating with 2.5 μM Rh123. The concentrations of the compounds that reach the maximum accumulation of Rh123 were set as 100% inhibition. Graphs represent the percentage inhibition (± SD of three independent experiments) compared to DMSO pre-treated cells. The half-maximal inhibitory concentration (IC50) values were calculated using nonlinear regression. C3N-Dbn-Trp2; black line (square): verapamil; blue line (triangle): tariquidar.

(b) Quantification of cell viability. PaKiT03 parental and *ABCB1* KO cells were pre-treated with 5 μM of C3N-Dbn-Trp2 (Dpn-Trp2) or verapamil for 30 minutes before adding 1 μM doxorubicin (Doxo). The cell viability was measured after 24 hours of Doxo treatment. Bar graphs represent the mean ± SD of three independent experiments. Unpaired two-tailed Student's t-test was performed for statistical analysis (***P<0.001). White bar: without Doxo; gray bar: with Doxo.

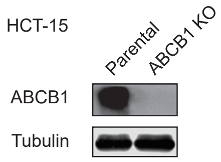

Supplementary Fig. S8 CRISPR *ABCB1* knockout of HCT-15 cells.

Western blot analysis of ABCB1 in HCT-15 parental and *ABCB1* knockout (KO) cells. Tubulin was used as a loading control.

Supplementary Fig S9. Images of Western blot membranes in Fig. 1a and 2e.

Fig. 1a

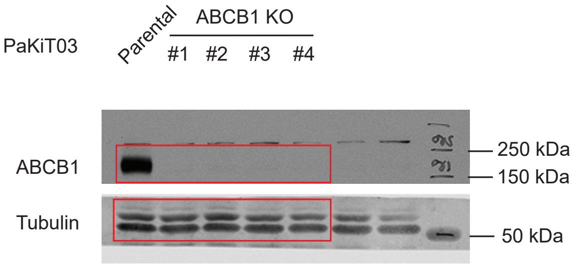

Fig. 2e

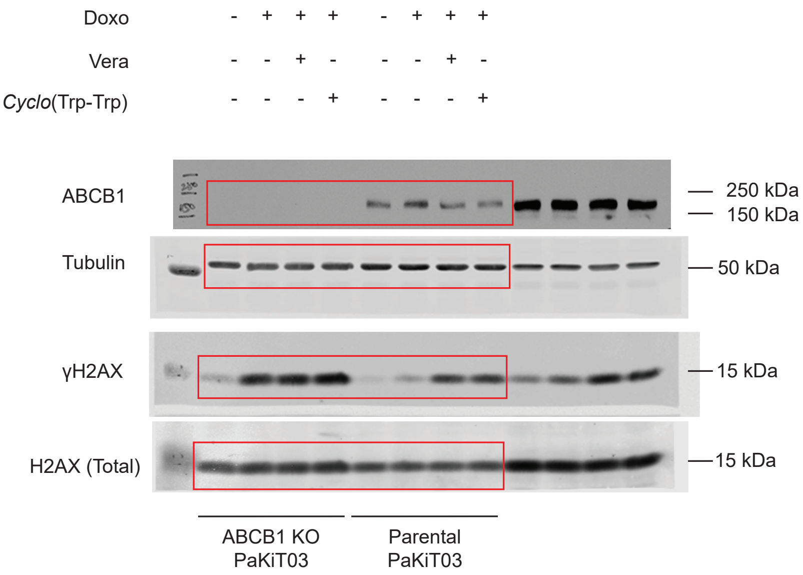

Supplementary Fig S10. Images of Western blot membranes in Fig. 3a and 3d.

Fig. 3a

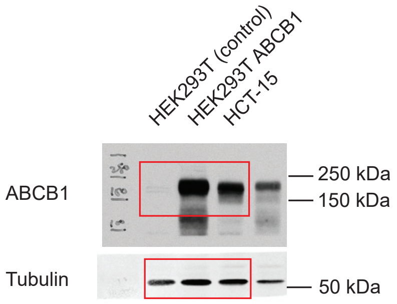

Fig. 3d

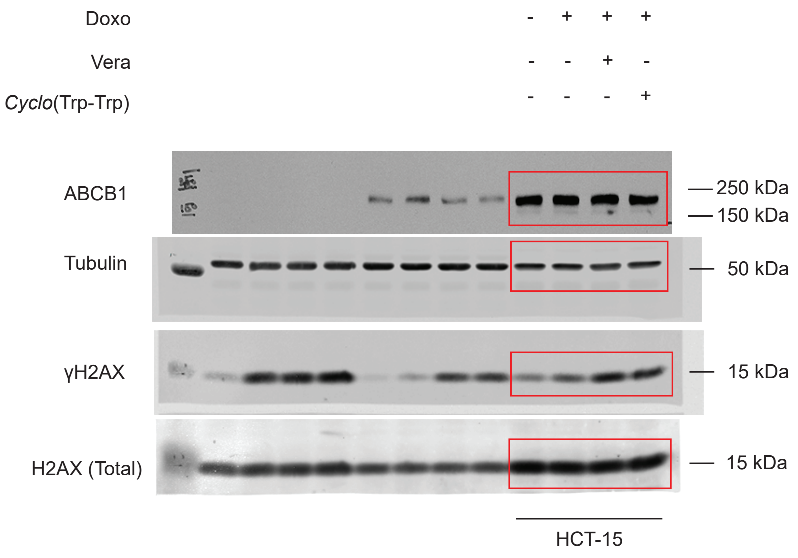

Supplementary Fig S11. Images of Western blot membranes in Fig. 8a.

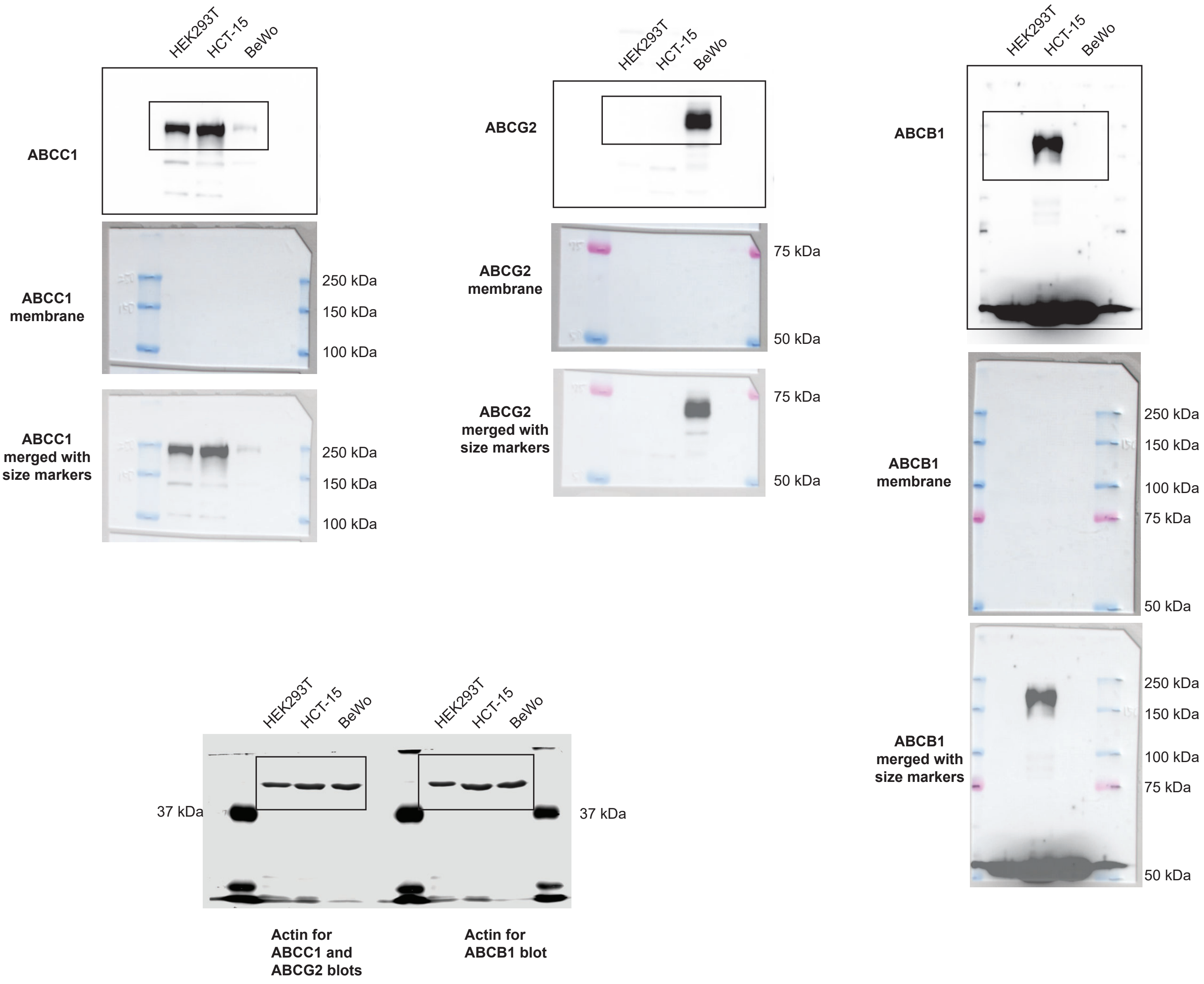

Supplementary Fig S12. Images of Western blot membranes in Fig. 8b, 8c, and 8f.

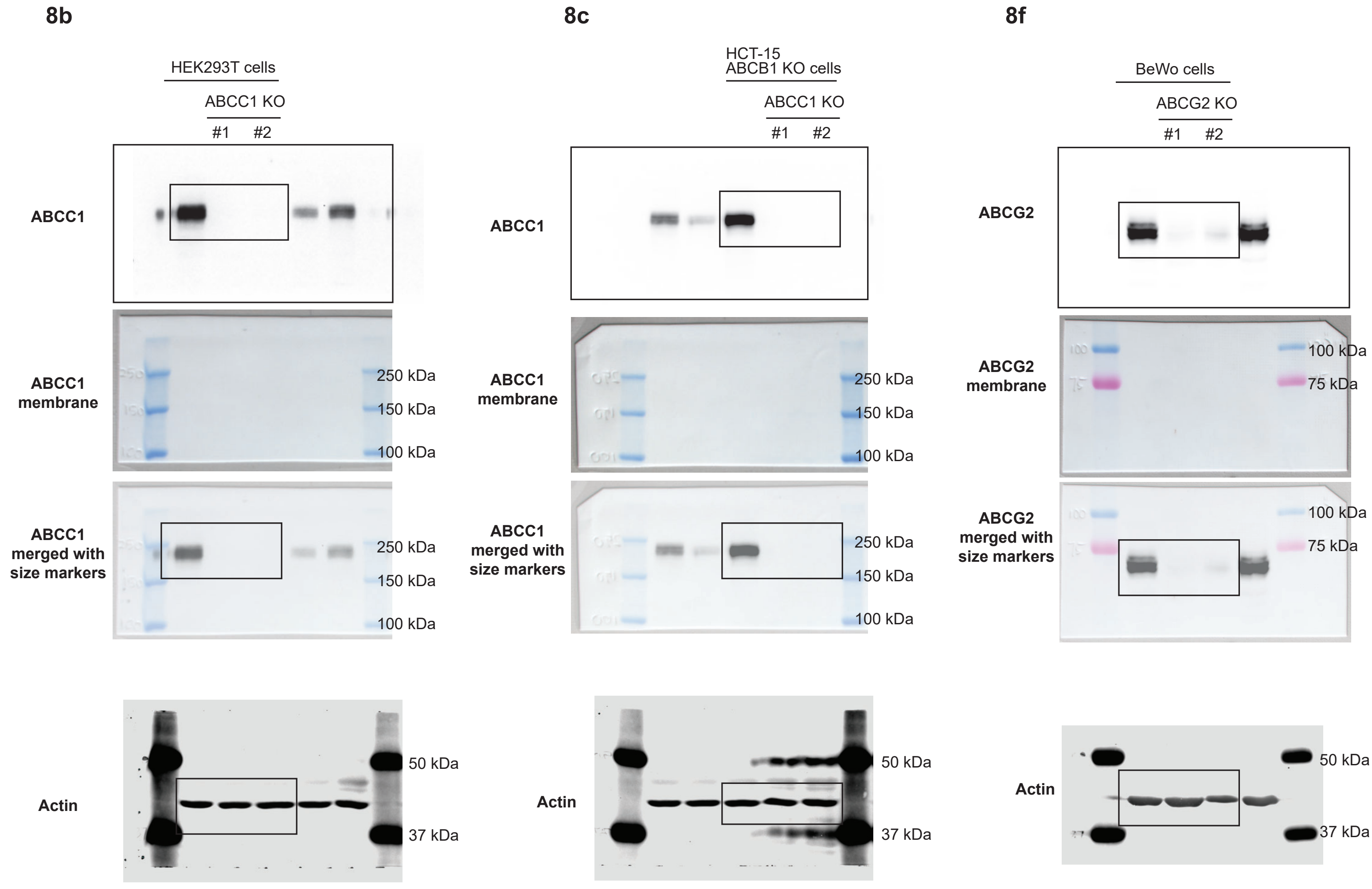

Supplementary Fig S13. Images of Western blot membranes in Supplementary Fig. S8.

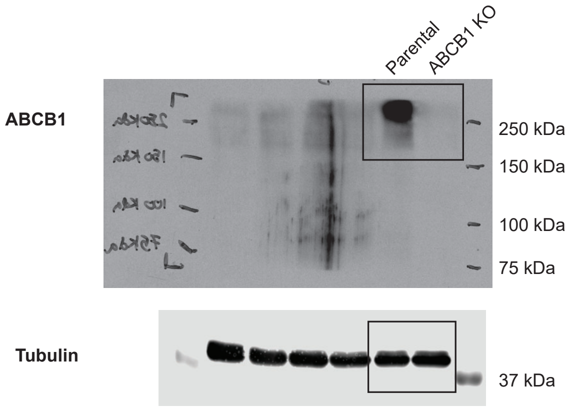

Supplementary Table S1: Molecular interactions of the compounds for truncated ABCB1 model during molecular dynamics simulations. In this system, a long loop that connects the N- and C-terminal domains in the cryo-electron microscopy structure has been truncated and capped. Comparable data with correct ranking of ligands was obtained for systems in which the missing loop was modelled. The mean number of hydrogen bonds and hydrophobic contacts between ligand and protein and between both ligands are shown, along with the binding energies based on the Molecular Mechanics Poisson-Boltzmann Surface Area (MM-PBSA) calculations.

System	Number of hydrogen bonds (protein-ligand)	Number of hydrogen bonds (ligand-ligand)	Number of hydrophobic contacts (protein-ligand)	Number of hydrophobic contacts (ligand-ligand)	total binding free energy (kcal/mol)
Cyclo-(L-Leu-L-Pro)	1.4 ± 1.0	0	22.9 ± 4.4	0.2 ± 0.5	-35.5 ± 1.5
Cyclo-(L-Trp-L-Trp)	3.9 ± 1.7	0	37.0 ± 5.0	1.7 ± 1.7	-42.6 ± 1.4
Cyclo-(L-1-methyl-Trp-L-1-methyl-Trp)	2.1 ± 1.1	0	41.0 ± 6.3	0.0 ± 0.0	-61.1 ± 1.5
C3N-Dbn-Trp2	1.1 ± 1.0	0	49.1 ± 5.4	4.0 ± 2.5	-68.1 ± 3.0

Supporting Information

For

Benzylation of cyclo-L-Trp-L-Trp-DKP

Table of Contents

Abbreviations 3
General methods 4
Scheme and experimental procedures for the synthesis of cyclo-L-Trp-L-Trp-DKP…………… 6
Scheme and general procedure for Benzylation of cyclo-L-Trp-L-Trp-DKP...............................10
Copies of Spectral Data.. 14

Abbreviations

AcOH	Acetic Acid
Boc anhydride	*tert*-butyloxycarbonyl anhydride
$CDCl_3$	Deuterated Chloroform
COSY	COrrelation SpectroscopY
DCM	Dichloromethane
DEPT	Distortionless Enhancement by Polarization Transfer
DKP	2,5-Diketopiperazine.
DMSO	Dimethyl Sulfoxide
EtOAc	Ethyl Acetate
HCl	Hydrochloric Acid
HMBC	Heteronuclear Multiple Bond Correlation
HOBt	N-hydroxybenzotriazole
HSQC	Heteronuclear Single Quantum Coherence
MeOH	Methanol
MHz	MegaHertz
mmol	Millimole
µmol	Micromole
Na_2CO_3	Sodium Carbonate
$NaHCO_3$	Sodium Bicarbonate
NaOAc	Sodium Acetate
Na_2SO_4	Sodium Sulphate

NMR	Nuclear Magnetic Resonance
NOESY	Nuclear Overhauser Effect SpectroscopY
pH	potenz hydrogen
PMA (stain)	Phosphomolybdic acid
ROESY	Rotating-frame nuclear Overhauser Effect SpectroscopY
RTet	Room temperature
TFA	Trifluoroacetic acid
THF	Tetrahydrofuran
TMS	Tetramethyl silane
Trp	Tryptophan
Trp-OMe	Tryptophan methyl ester

General methods

Reagents, Solvents and Glassware

Unless otherwise stated all reactions were carried out under a blanket of nitrogen or under regular atmospheric conditions, using standard syringe-septum, and cannulation techniques. KOH, NaOH, Na_2CO_3, $NaHCO_3$, Na_2SO_4, and were purchased from Merck. Boc anhydride, HOBT, NaOAc, $SOCl_2$, TFA, p-toluene sulfonic acid, triethyl amine were purchased from Spectrochem chemicals. L- and D-Tryptophan were purchased from TCI chemicals. Glacial acetic acid was obtained from Rankem and used without further purification. Dry THF and dichloromethane were purchased from Sigma Aldrich and used without further purification. All other solvents were purified according to specific literature procedures, unless otherwise noted.

Chromatography

Thin-layer chromatography (TLC) was performed using silica gel 60 GF_{254} pre-coated aluminium backed plates (2.5 mm), specifically to monitor the progress of each chemical reaction and used as a guide for purification of the ensuing mixtures. Various combinations of ethyl acetate/hexanes and methanol/DCM were used as eluent. Visualization of spots after TLC was accomplished by exposure to staining agents (iodine vapour, ninhydrin and /or PMA) and/or UV light (254 nm). All compounds were purified using gravity column chromatography (Silica gel grade: 200-400 mesh, 40-63 µm). Yields refer to compounds isolated to analytical purity after chromatography.

Analytical Characterization

NMR spectroscopic analyses (^{1}H NMR, ^{13}C NMR and 2D NMR) were conducted for all new compounds. ^{1}H (400 MHz), ^{13}C (101 MHz), and 2D-NMR (COSY, NOESY, ROESY, HSQC and HMBC) spectra were recorded on a 400 MHz spectrometer. Pertinent frequency is specifically reported for each compound. Chemical shift values (δ) for NMR spectra are reported in parts per million (ppm) relative to the residual (indicated) solvent peak ($CDCl_3$, DMSO-D_6 or CD_3OD). Additional peaks other than the compound in question, if any, are calibrated based on reported values for trace impurities. Coupling constants are reported in Hz. Data for ^{1}H NMR are reported as follows: chemical shift (δ, ppm), multiplicity (s = singlet, brs = broad singlet, d = doublet, t = triplet, q = quartet, ddd = double double doublet, m = multiplet, cm = complex multiplet), integration corresponding to the number of protons followed by coupling constants in Hz. For ^{13}C NMR spectra, the nature of the carbons (C, CH, CH_2 or CH_3) was determined by recording the Distortionless Enhancement by Polarization Transfer (DEPT) experiment, and notations are provided in parentheses.^{13}C NMR data is reported in parts per

million (δ) relative to the residual (indicated) solvent peak. Chiroptical measurements ([α]D) were obtained on a polarimeter in a 100 × 2 mm cell. Mass samples were analysed by High-resolution mass spectrometry using ESI TOF.

Scheme and experimental procedures for the synthesis of cyclo-L-Trp-L-Trp-DKP

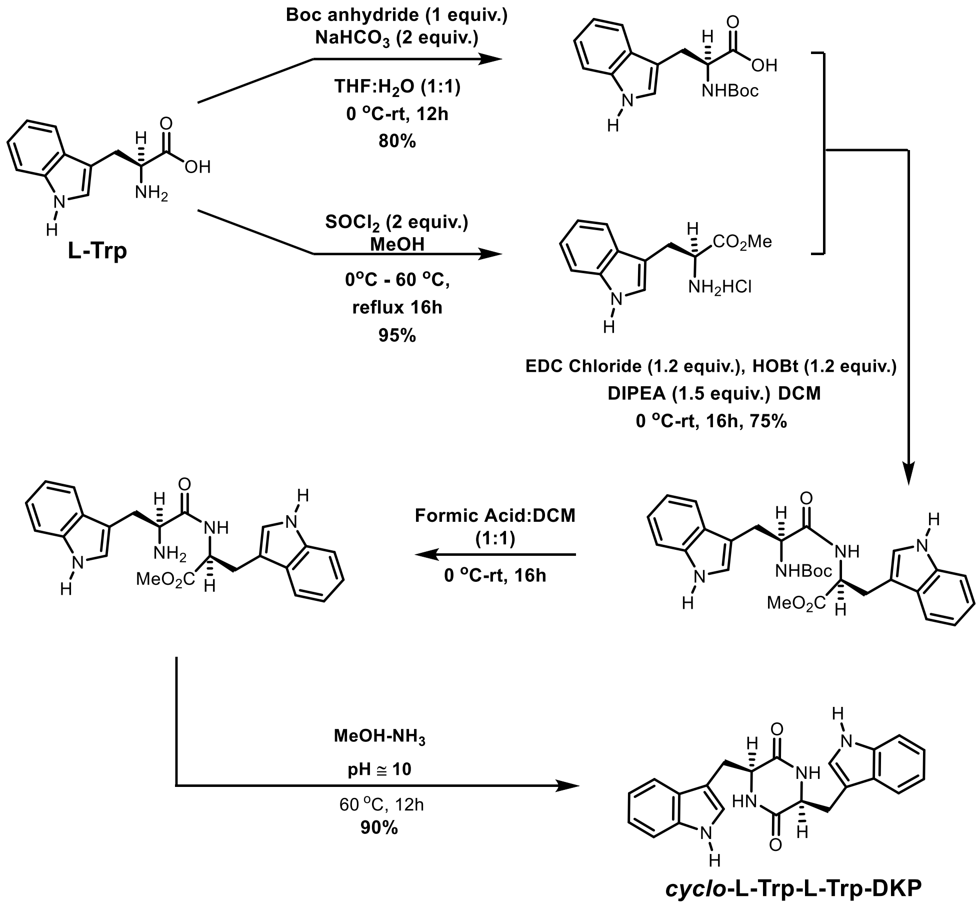

Scheme S1: Synthesis of *cyclo*-L-Trp-L-Trp DKP

L-Trp methyl ester hydrochloride (*L*-Trp-OMe-HCl):

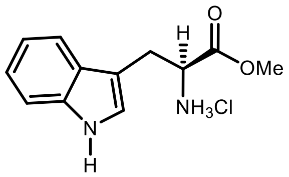

1

Thionyl chloride (9 mL, 124 mmol, 2.5 equiv.) was added drop wise to an ice-cold (0 °C) solution of L -Trp (10.0 g, 48.96 mmol, 1.0 equiv.) in anhydrous methanol (excess) under magnetic stirring. The solution was stirred at 0 °C for 30 min. and then heated at 60 °C for 18

h. After evaporation of methanol *in vacuo*, a white residue of hydrochloride salt was obtained, which was used without any purification (12.5g, 48.96 mmol). *Data*. **^{1}H NMR** (400 MHz, CD_3OD) δ 7.54 (dt, *J* = 7.8, 1.0 Hz, 1H), 7.40 (dt, *J* = 8.1, 0.9 Hz, 1H), 7.20 (s, 1H), 7.15 (ddd, *J* = 8.2, 7.1, 1.2 Hz, 1H), 7.07 (ddd, *J* = 8.0, 7.0, 1.1 Hz, 1H), 4.33 (dd, *J* = 7.4, 5.5 Hz, 1H), 3.80 (s, 3H), 3.52 – 3.41 (m, 1H), 3.36 (dd, *J* = 15.2, 7.4 Hz, 1H). **^{13}C NMR** δ (101 MHz, CD_3OD) 169.4, 136.9, 126.8, 124.2, 121.6, 118.9, 117.4, 111.3, 106.1, 53.2, 52.3, 26.2. **HRMS**, ESI, ($M+H^+$): m/z calcd. for $C_{12}H_{15}N_2O_2$ 219.1128, found 219.1127.

***N*$^{\alpha}$-Boc-*L*-Trp-acid**

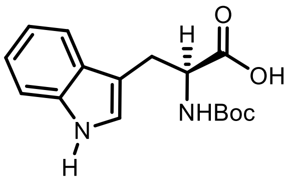

2

To a magnetically stirred clear solution of L-Trp (4 g, 19.6 mmol, 1.0 equiv.) in 200 mL of THF-H_2O (1:1) was added Na_2CO_3 (4.9 g, 58.76 mmol, 3.0 equiv.) and $NaHCO_3$ (6.24 g, 58.76 mmol, 3.0 equiv). The resulting turbid solution was cooled to 0 °C (H_2O/ice bath) and stirred for 15 min. To this mixture was added Boc Anhydride (4.5 mL, 19.6 mmol, 1.0 equiv.) drop-wise. The resulting solution was stirred for 15-20 min at 0 °C, the ice bath was removed and reaction was stirred at r.t. overnight. THF was evaporated on rotary evaporation and the crude was diluted with EtOAc (100 mL). This mixture was acidified by addition of a 1N aq. HCl solution. Subsequently, it was transferred to a separating funnel, the organic layer was washed with brine and dried over anhydrous Na_2SO_4. Concentration under reduced pressure gave 5.7 g, (96% yield, 18.73 mmol) as a white solid which was directly used for the next step without further purification. *Data:*. **^{1}H NMR** (400 MHz, DMSO-*d*$_6$) δ 12.53 (s, 1H), 10.82 (s, 1H), 7.52 (d, *J* = 7.9 Hz, 1H), 7.33 (d, *J* = 8.1 Hz, 1H), 7.14 (d, *J* = 2.0 Hz, 1H), 7.08 – 7.04 (m, 1H), 7.00 – 6.95 (m, 2H), 4.17-4.11 (m, 1H), 3.13 (dd, *J* = 14.6, 4.8 Hz, 1H), 2.97 (dd, *J* = 14.6, 9.3 Hz, 1H), 1.33 (s, 9H); **^{13}C NMR** (101 MHz, DMSO-*d*$_6$) δ 174.0, 155.4, 136.1, 127.2, 123.6, 120.9, 118.33, 118.13, 111.38, 110.16, 78.0, 54.50, 28.2, 26.8.

methyl (tert-butoxycarbonyl)-L-tryptophyl-L-tryptophanate

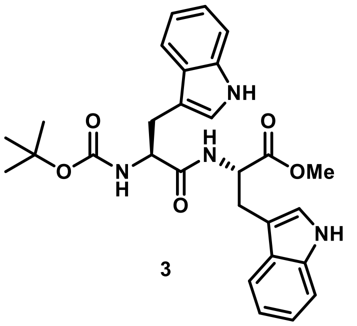

3

N$^{\alpha}$-Boc-L-Trp-acid (**2**) (5 g, 16.43 mmol, 1 equiv.) and L-Trp-OMe-HCl(**1**) (7.17 g, 32.86 mmol, 2 equiv.) were suspended in anhydrous dichloromethane (250 mL). The suspension was cooled to 0 °C in an ice-water bath. This was followed by addition of *N,N*-diisopropylethylamine (5.7 mL, 32.86 mmol, 2.00 equiv.) which resulted in a clear solution. Subsequently, N-Hydroxybenzotriazole monohydrate (2.77 g, 18.07 mmol, 1.1 equiv.) was added to the reaction mixture, followed by the addition of 1-Ethyl-3-(3-dimethylaminopropyl)carbodiimide hydrochloride (3.78 g, 19.71 mmol, 1.2 equiv.). The reaction was allowed to stir at 0 °C for one hour and then at room temperature for fifteen hours. The reaction mixture was washed with saturated citric acid solution in a separatory funnel, followed by washing with saturated sodium bicarbonate solution. The organic layer was dried over anhydrous sodium sulphate and was evaporated *in vacuo*. The compound was purified using column chromatography with 35-40% EtOAc/Hexanes as eluent. M.p.187-188 °C.(5.64 g, 68% yield, 11.17 mmol). **^{1}H NMR** (500 MHz, DMSO-d_6) δ 10.89 (d, *J* = 1.8 Hz, 1H), 10.81 (s, 1H), 8.27 (d, *J* = 7.4 Hz, 1H), 7.59 (d, *J* = 7.9 Hz, 1H), 7.49 (d, *J* = 7.9 Hz, 1H), 7.34 (ddd, *J* = 11.2, 5.5, 4.5 Hz, 2H), 7.18 (d, *J* = 2.1 Hz, 1H), 7.10 – 7.03 (m, 3H), 7.01 – 6.95 (m, 2H), 6.73 (d, *J* = 8.4 Hz, 1H), 4.58 (dd, *J* = 13.8, 7.1 Hz, 1H), 4.24 (ddd, *J* = 28.3, 16.5, 11.9 Hz, 1H), 3.56 (s, 3H), 3.19 – 3.02 (m, 3H), 2.88 (dd, *J* = 14.7, 9.5 Hz, 1H), 1.30 (s, 9H). **^{13}C NMR** (101 MHz, DMSO-d_6) δ 173.8 (C=O), 172.2 (C=O), 155.1 (carbamate C=O), 136.1 (C), 136.0 (C), 127.4 (C), 127.1 (C), 123.7 (CH), 123.6 (CH), 121.0 (CH), 120.8 (CH), 118.5 (CH), 118.4 (CH), 118.1 (CH), 118.0 (CH), 111.4 (CH), 111.2 (CH), 110.1 (C), 109.2 (C), 78.1 (C), 55.0 (CH), 53.0 (CH), 51.8 (CH_3), 29.0 (3 X CH_3), 27.7 (CH_2), 27.1 (CH_2).

Methyl L-tryptophyl-L-tryptophanate.formic acid salt

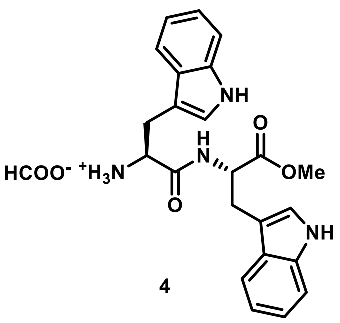

4

To the magnetically stirred solution of methyl (tert-butoxycarbonyl)-L-tryptophyl-L-tryptophanate(**3)** (5.64 g, 11.17 mmol, 1.00 equiv.) in 50 mL of dichloromethane was added 50 mL of formic acid dropwise at room temperature. The reaction was allowed to stir overnight for 12 hours. Subsequently, after the completion of the reaction dichloromethane and formic acid were evaporated *in vacuo*, resulting in a reddish oil which was used for next step without further purification.

(3*S*,6*S*)-3,6-bis((1H-indol-3-yl)methyl)piperazine-2,5-dione (*cyclo*-L-Trp-L-Trp DKP):

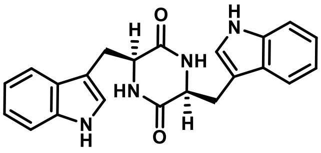

5

A homogenous solution (under stirring with a magnetic bar) of amine(**4**) (2.50 g; 6.18 mmol, 1.00 equiv.) was refluxed at 60 °C overnight in 14 M methanolic ammonia (pH ≈ 10, excess). The solvent was evaporated under reduced pressure. The resulting residue was washed with ice-cold water and sonicated till suspension. The suspension was vacuum filtered and the solid was washed with ice-cold acetonitrile (10 mL x 3) which furnished pure ***cyclo*-L-Trp-L-Trp DKP** as a white solid (88%, 3.66 g, 9.84 mmol from **3**). **^{1}H NMR** (400 MHz, DMSO-*d*$_6$) δ 10.85 (d, *J* = 2.4 Hz, 2H), 7.72 (d, *J* = 2.7 Hz, 2H), 7.36 (d, *J* = 7.9 Hz, 2H), 7.29 (d, *J* = 8.0 Hz, 2H), 7.05 (ddd, *J* = 8.0, 6.9, 1.2 Hz, 2H), 6.96 (ddd, *J* = 7.9, 7.0, 1.0 Hz, 2H), 6.60 (d, *J* = 2.2 Hz, 2H), 3.87 (dt, *J* = 6.7, 3.3 Hz, 2H), 2.71 (dd, *J* = 14.3, 4.2 Hz, 2H), 2.18 (dd, *J* = 14.3, 6.6 Hz, 2H). **^{13}C NMR** (101 MHz, DMSO-*d*$_6$) δ 165.1 (C=O), 134.4 (C), 125.7 (C), 122.8 (CH), 119.2 (CH), 116.9 (CH), 116.7 (CH), 109.6 (CH), 107.1 (C), 53.6 (CH), 28.3 (CH$_2$). **HRMS**, EI, $(M+H)^+$: m/z calcd. for $C_{22}H_{21}N_4O_2$ 373.1659, found 373.1661.

Scheme and experimental procedure for benzylation of cyclo- L-trp-L-Trp-DKP

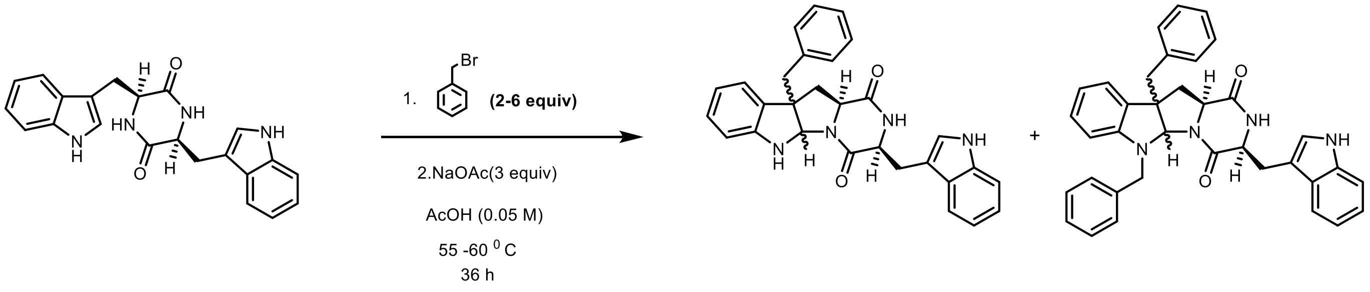

Scheme S2: benzylation of *cyclo*- L-trp-L-Trp-DKP

To the magnetically stirred solution of cyclo-L-Trp-L-Trp DKP (1g, 2.68. mmol, 1.00 equiv.) in 50 mL acetic acid sodium acetate (659.5 mg, 8.04 µmol, 3.00 equiv.) was added and then benzyl bromide (1.90mL, 16.08 mmol, 6 equiv.) was added portion wise 2 equiv at a time at room temperature. The reaction mixture was allowed to stirrer overnight at 55°C (using oil bath). After completion of reaction, the acetic acid was evaporated in vacuo. The resultant residue was purified by column chromatography (DCM/MeOH 98:2) followed by preparative TLC to yield C3-benzylated cyclo-L-Trp-L-Trp and its bis-C3-N-indole- debenzylated cyclo-L-Trp-L-Trp, in 38% overall yield, and in 1:3 ratio.

(3S,11aS)-3-((1H-indol-3-yl)methyl)-6,10b-dibenzyl-2,3,6,10b,11,11a-hexahydro-4H-pyrazino[1',2':1,5]pyrrolo[2,3-b]indole-1,4(5aH)-dione(Di-benzylated endo-pyrroloindoline)

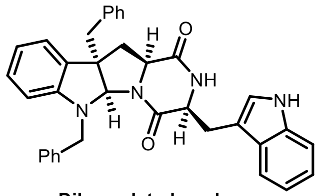

Dibenzylated endo-pyrroloindoline

The compound, Di-benzylated endo-pyrroloindoline was synthesized according to general procedure for benzylation of cyclo-L-Trp-L-Trp-DKP mentioned above. (Cream coloured, solid powder)

Data: $[\alpha]\frac{20}{D}$: -17.30 (c 0.5 , CH_3OH) **^{1}HNMR** (400 MHz, Chloroform-*d*) δ 8.02 (s, 1H), 7.48 (dd, *J* = 7.9, 1.0 Hz, 1H), 7.34 (dt, *J* = 8.2, 1.0 Hz, 1H), 7.31 – 7.24 (m, 5H), 7.24 – 7.19 (m, 4H), 7.17 – 7.12 (m, 4H), 7.11 – 7.08 (m, 1H), 6.88 (d, *J* = 2.3 Hz, 1H), 6.77 (td, *J* = 7.5, 1.0 Hz, 1H), 6.40 (d, *J* = 7.9 Hz, 1H), 5.88 (s, 1H), 5.40 (s, 1H), 4.93 (d, *J* = 16.5 Hz, 1H), 4.61 (d, *J* = 16.5 Hz, 1H), 3.98 – 3.84 (m, 1H), 3.49 (ddd, *J* = 15.1, 3.9, 1.1 Hz, 1H), 3.26 (dd, *J* = 11.2, 7.2 Hz, 1H), 3.18 (d, *J* = 13.8 Hz, 1H), 3.10 (d, *J* = 13.7 Hz, 1H), 2.60 – 2.50 (m, 2H), 2.40 (dd, *J* = 13.4, 11.2 Hz, 1H). **^{13}C NMR** (101 MHz, $CDCl_3$) δ 169.7, 168.4, 148.9, 139.5, 136.7, 136.6, 133.1, 130.1, 129.0, 128.8, 128.5, 127.4, 127.2, 126.8, 123.3, 122.9, 122.1, 120.1, 118.4, 118.4, 111.7, 109.8, 107.6, 85.8, 58.8, 55.9, 55.0, 52.3, 42.3, 39.1, 26.0**.** **HRMS,** ESI ($M+H^+$): m/z calcd for $C_{36}H_{33}N_4O_2$ 553.2589, found 553.2581

(3S,11aS)-3-((1H-indol-3-yl)methyl)-10b-benzyl-2,3,6,10b,11,11a-hexahydro-4H-pyrazino[1',2':1,5]pyrrolo[2,3-b]indole-1,4(5aH)-dione (**mono-benzylated endo-pyrroloindoline)**

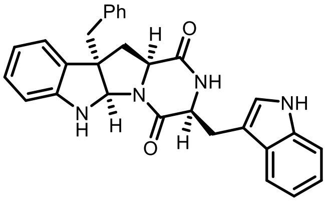

mono-benzylated endo-pyrroloindoline

The compound mono-benzylated endo-pyrroloindoline was synthesized according to the general procedure for benzylation of cyclo-L-Trp-L-Trp-DKP as mentioned above. (Yellowish ,solid powder)

Data: $[\alpha]\frac{20}{D}$ +19.14 (c 0.035 ,CH_3OH) **^{1}H NMR** (400 MHz, Chloroform-*d*) δ 8.09 (s, 2H), 7.54 (d, *J* = 8.0 Hz, 3H), 7.36 (d, *J* = 8.2 Hz, 3H), 7.32 – 7.19 (m, 12H), 7.18 – 7.05 (m, 9H), 7.02 (d, *J* = 2.4 Hz, 2H), 6.79 (d, *J* = 1.0 Hz, 1H), 6.61 (s, 1H), 5.61 (d, *J* = 1.9 Hz, 2H), 5.30 (s, 0H), 4.20 (ddd, *J* = 10.9, 3.8, 1.5 Hz, 2H), 3.80 – 3.56 (m, 4H), 3.22 – 2.95 (m, 4H), 2.65 (dd, *J* = 13.4, 8.0 Hz, 2H), 2.46 (dd, *J* = 13.4, 9.3 Hz, 2H). **^{13}C NMR** (101 MHz, $CDCl_3$) δ 169.0, 167.4, 147.5, 136.8, 136.7, 132.6, 130.3, 128.8, 128.7, 127.3, 125.9, 123.6, 123.4, 123.0,

120.2, 119.4, 118.5, 111.7, 111.3, 109.7, 81.0, 58.0, 56.2, 54.6, 43.1, 38.6, 26.7. **HRMS,** ESI ($M+H^+$) m/z calcd for $C_{29}H_{27}N_4O_2$ 463.2129, found 463.2113

(3S,11aS)-3-((1H-indol-3-yl)methyl)-6,10b-dibenzyl-2,3,6,10b,11,11a-hexahydro-4H-pyrazino[1',2':1,5]pyrrolo[2,3-b]indole-1,4(5aH)-dione(**C3N-DBn-Trp2**)

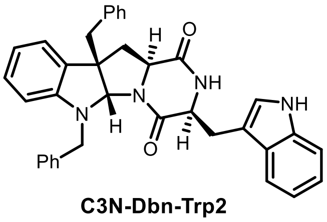

C3N-Dbn-Trp2

The compound C3N-DBn-Trp2 was synthesized according to the general procedure for benzylation of cyclo-L-Trp-L-Trp-DKP as mentioned above. (Solid powder, cream-coloured).

Data: $[\alpha]\frac{20}{D}$ – 214.5 (c 0.1 , CH_3OH) **^{1}H NMR** (400 MHz, Chloroform-*d*) δ 8.34 – 7.99 (m, 1H), 7.63 (dd, *J* = 7.7, 1.3 Hz, 1H), 7.39 (dt, *J* = 8.2, 0.8 Hz, 1H), 7.31 – 7.07 (m, 10H), 7.09 – 6.92 (m, 4H), 6.77 (dt, *J* = 8.4, 1.8 Hz, 3H), 6.63 (td, *J* = 7.4, 0.9 Hz, 1H), 6.17 (d, *J* = 7.8 Hz, 1H), 5.99 (s, 1H), 5.55 (s, 1H), 4.69 (d, *J* = 16.4 Hz, 1H), 4.44 (d, *J* = 16.3 Hz, 1H), 4.28 (ddt, *J* = 8.0, 3.5, 1.4 Hz, 1H), 4.03 – 3.87 (m, 1H), 3.41 (dd, *J* = 3.9, 0.9 Hz, 1H), 3.21 (dd, *J* = 14.8, 8.0 Hz, 1H), 2.72 (d, *J* = 13.3 Hz, 1H), 2.47 (dd, *J* = 12.0, 5.5 Hz, 1H), 2.36 (d, *J* = 13.3 Hz, 1H), 1.52 (t, *J* = 11.9 Hz, 1H). **^{13}C NMR** (101 MHz, $CDCl_3$) δ 168.2, 165.0, 150.5, 138.8, 136.7, 136.5, 130.3, 130.3, 129.1, 128.5, 128.2, 127.0, 127.0, 126.9, 126.8, 124.1, 124.0, 123.0, 120.3, 119.2, 117.8, 111.6, 109.4, 106.5, 83.5, 58.5, 55.3, 55.2, 49.5, 44.2, 41.9, 29.1. **HRMS,** ESI, ($M+H^+$) m/z calcd for $C_{36}H_{33}N_4O_2$ 553.2589, found 553.2577

(3S,11aS)-3-((1H-indol-3-yl)methyl)-10b-benzyl-2,3,6,10b,11,11a-hexahydro-4H-pyrazino[1',2':1,5]pyrrolo[2,3-b]indole-1,4(5aH)-dione(**mono-benzylated-exo-pyrroloindoline**)

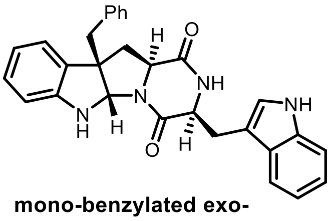

mono-benzylated exo-pyrroloindoline

The compound mono-benzylated exo-pyrroloindoline compound was synthesized according to the general method for benzylation of cyclo-L-Trp-L-Trp-DKP as mentioned above. (Solid powder, yellowish).

Data: $[\alpha]\frac{\mathbf{20}}{\boldsymbol{D}}$ – 192.83 (c 0.06 ,CH_3OH) **^{1}H NMR** (400 MHz, Chloroform-*d*) δ 8.16 (s, 1H), 7.55 (d, *J* = 7.9 Hz, 1H), 7.38 (dt, *J* = 8.2, 0.9 Hz, 1H), 7.30 – 7.18 (m, 5H), 7.17 – 7.04 (m, 2H), 7.01 (dd, *J* = 6.3, 3.2 Hz, 2H), 6.82 – 6.68 (m, 2H), 6.60 (dt, *J* = 7.8, 0.8 Hz, 1H), 5.70 (s, 1H), 5.39 (s, 1H), 5.02 (s, 1H), 4.30 (ddd, *J* = 10.4, 3.8, 1.8 Hz, 1H), 3.93 (ddd, *J* = 11.3, 5.9, 1.8 Hz, 1H), 3.82 – 3.56 (m, 1H), 2.99 (dd, *J* = 14.8, 10.4 Hz, 1H), 2.96 (d, *J* = 13.6 Hz, 1H), 2.88 (d, *J* = 13.4 Hz, 1H) **^{13}C NMR** (101 MHz, $CDCl_3$) δ 168.9, 166.2, 149.2, 136.7, 136.6, 130.5, 130.3, 129.1, 128.3, 127.0, 126.8, 124.1, 123.5, 123.0, 120.3, 119.4, 118.7, 111.7, 109.8, 109.7, 79.7, 59.2, 56.3, 54.8, 43.1, 38.3, 27.3. HRMS, ESI, $(M+H^+)$ m/z calcd for $C_{29}H_{27}N_4O_2$ 463.2119 found 463.2116

Copies of Spectral Data

^{1}H-NMR ,^{13}C-NMR, HRMS,

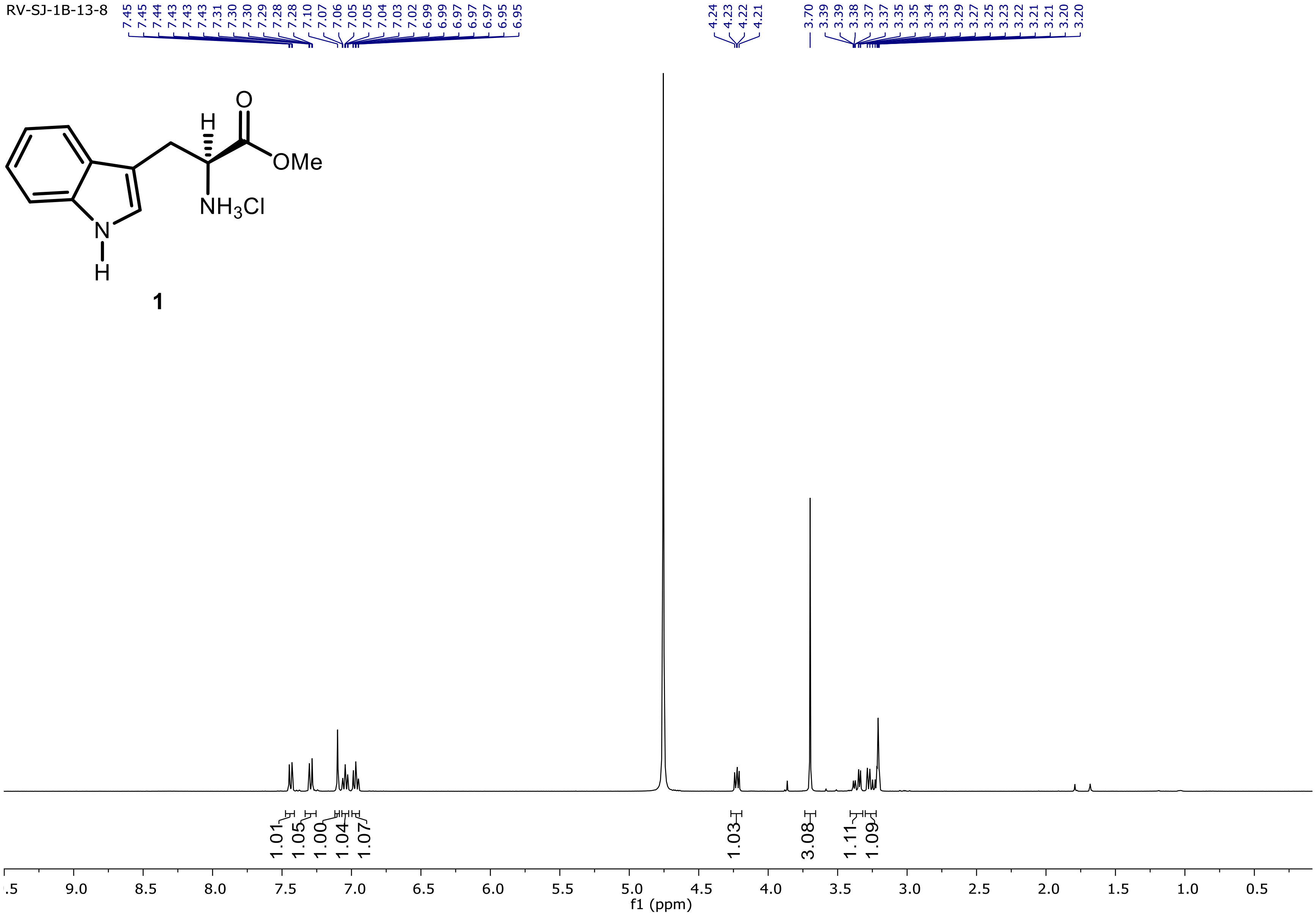
RV-SJ-1B-13-8
7.45 7.45 7.44 7.43 7.43 7.43 7.31 7.30 7.30 7.29 7.28 7.28 7.10 7.07 7.06 7.05 7.05 7.04 7.03 7.02 6.99 6.97 6.97 6.95 6.95
4.24 4.23 4.22 4.21
3.70 3.39 3.39 3.38 3.37 3.37 3.35 3.35 3.34 3.33 3.29 3.27 3.25 3.23 3.22 3.21 3.21 3.20 3.20
O
H
OMe
NH3Cl
N
H
1
1.01 1.05 1.00 1.04 1.07
1.03
3.08
1.11 1.09
.5 9.0 8.5 8.0 7.5 7.0 6.5 6.0 5.5 5.0 4.5 4.0 3.5 3.0 2.5 2.0 1.5 1.0 0.5
f1 (ppm)

RV-SJ-1B-13-8

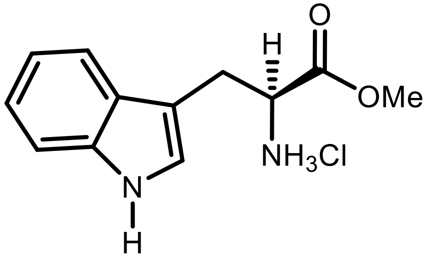
O
H
OMe
NH3Cl
N
H

1

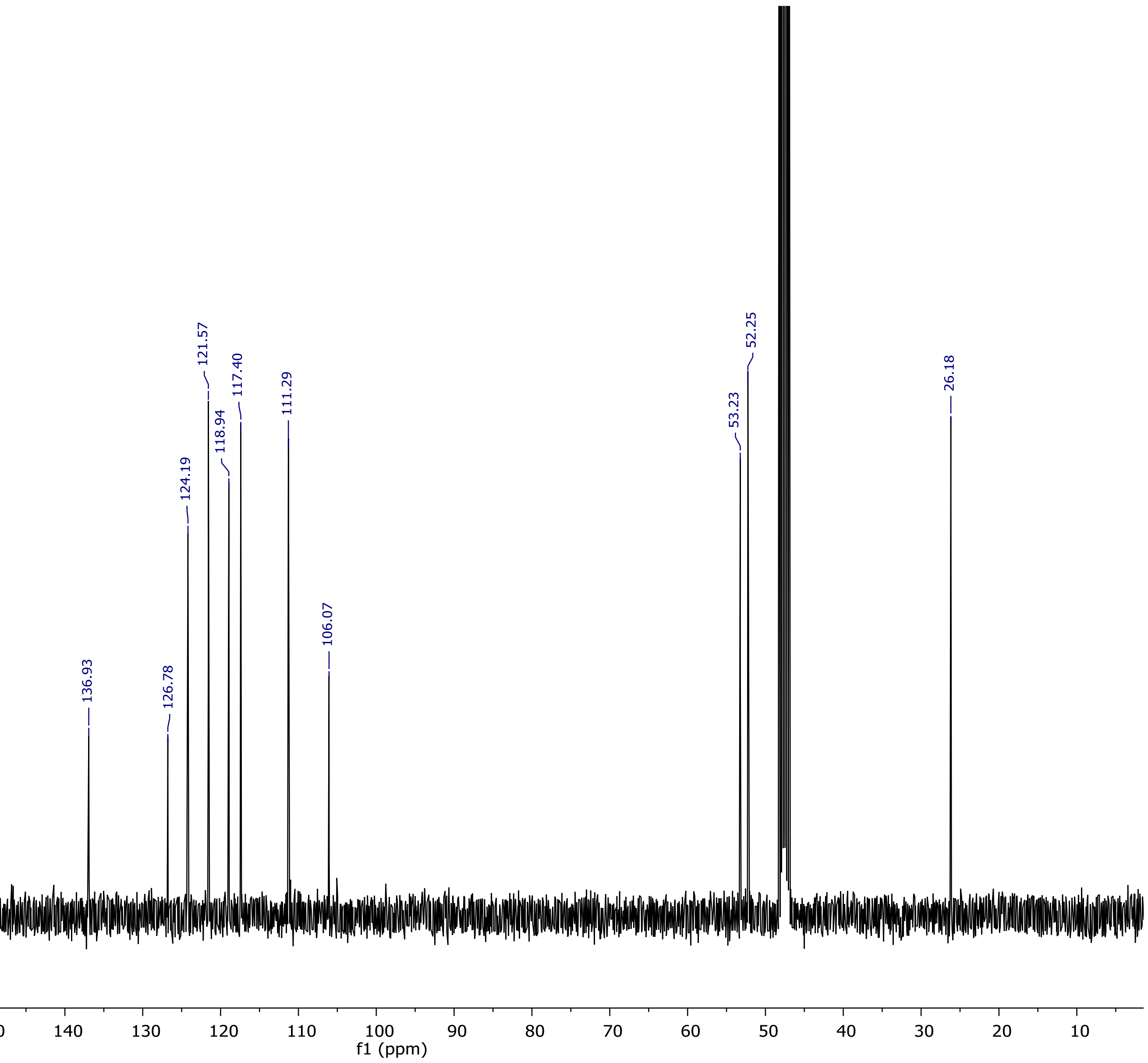
169.42
136.93
126.78
124.19
121.57
118.94
117.40
111.29
106.07
53.23
52.25
26.18
190 180 170 160 150 140 130 120 110 100 90 80 70 60 50 40 30 20 10
f1 (ppm)

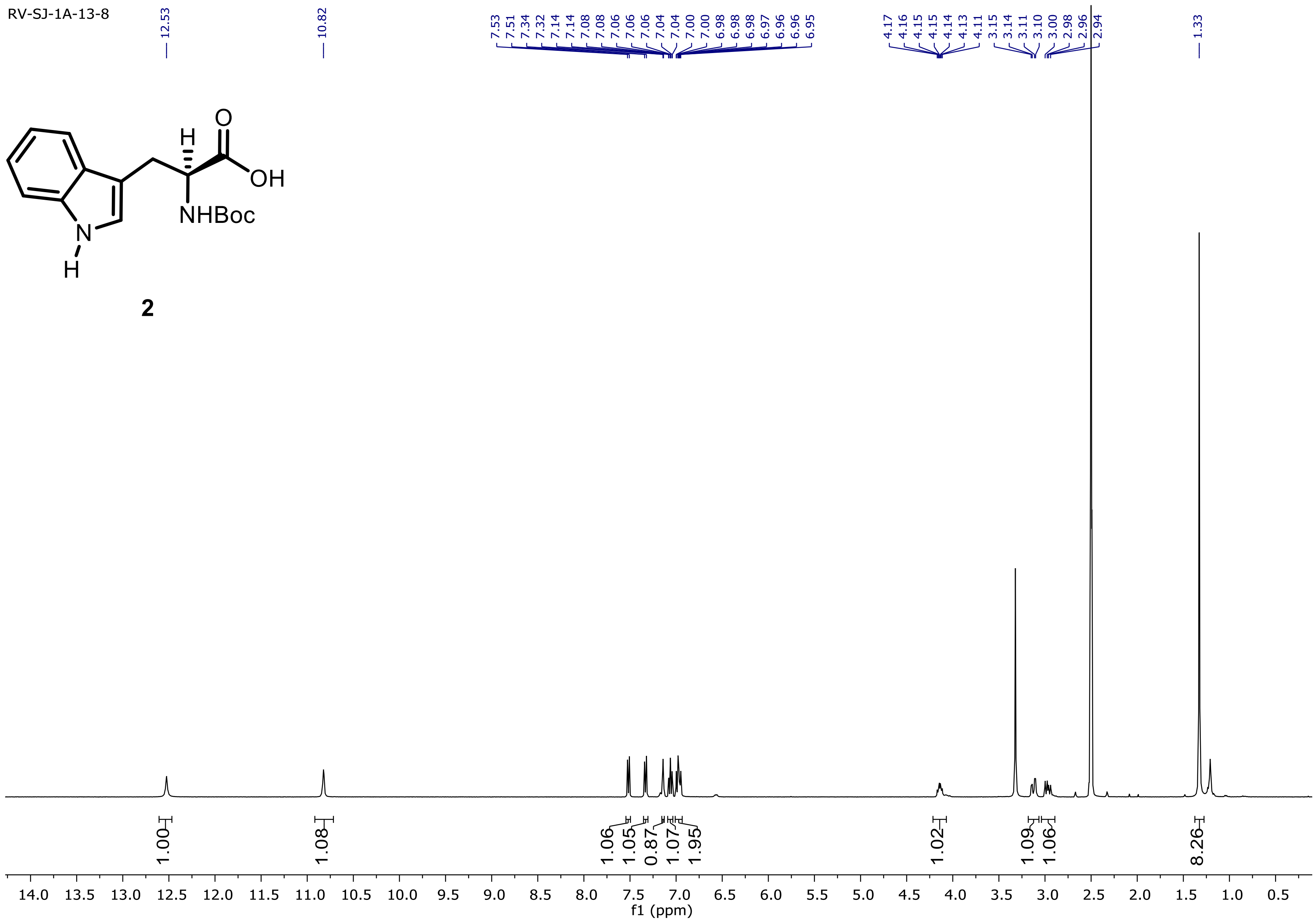
RV-SJ-1A-13-8
12.53
10.82
7.53 7.51 7.34 7.32 7.14 7.08 7.08 7.06 7.06 7.06 7.04 7.04 7.00 7.00 6.98 6.98 6.98 6.97 6.96 6.96 6.95
4.17 4.16 4.15 4.15 4.14 4.13 4.11 3.15 3.14 3.11 3.10 3.00 2.98 2.96 2.94
1.33
H
O
OH
NHBoc
N
H
2
1.00
1.08
1.06
1.05
0.87
1.07
1.95
1.02
1.09
1.06
8.26
14.0 13.5 13.0 12.5 12.0 11.5 11.0 10.5 10.0 9.5 9.0 8.5 8.0 7.5 7.0 6.5 6.0 5.5 5.0 4.5 4.0 3.5 3.0 2.5 2.0 1.5 1.0 0.5
f1 (ppm)

RV-SJ-1A-13-8

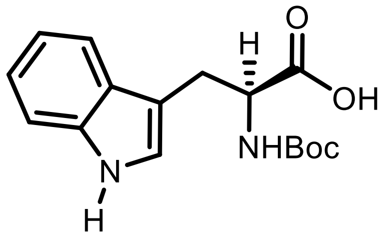

2

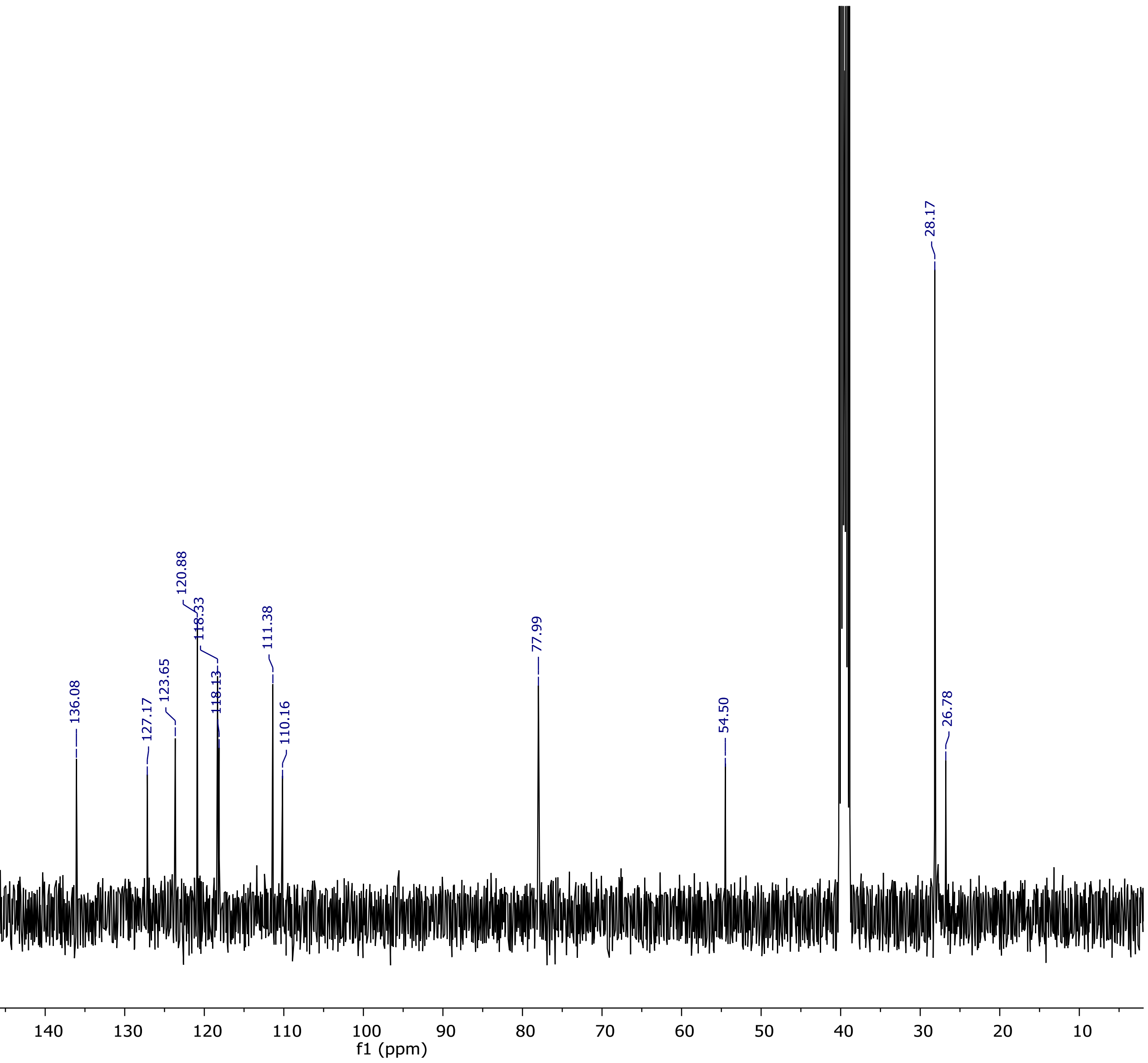
173.95
155.40
136.08
127.17
123.65
120.88
118.33
118.13
111.38
110.16
77.99
54.50
28.17
26.78
190
180
170
160
150
140
130
120
110
100
90
80
70
60
50
40
30
20
10
f1 (ppm)

RV-SJ-EDCL

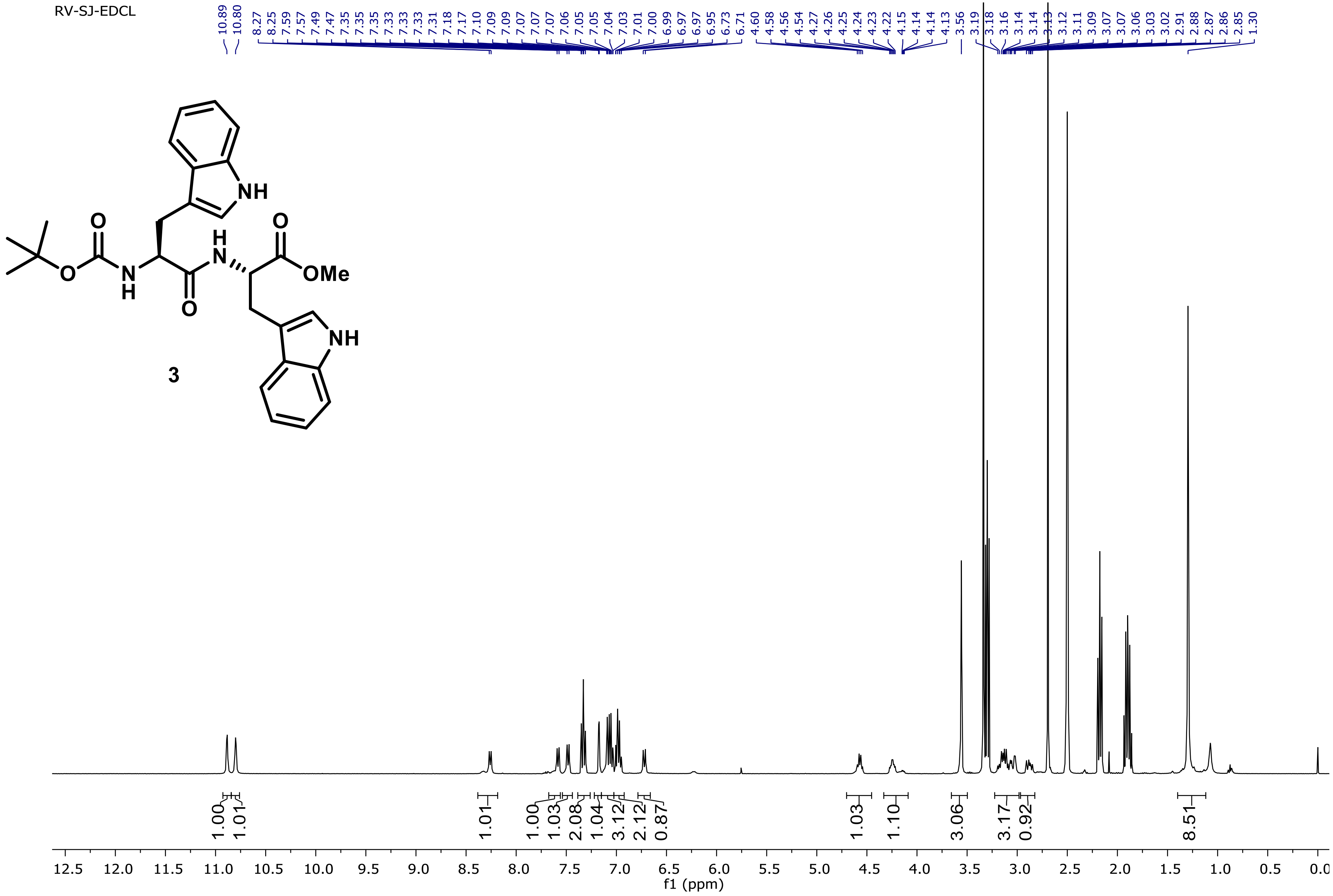

RV-SJ-EDCL

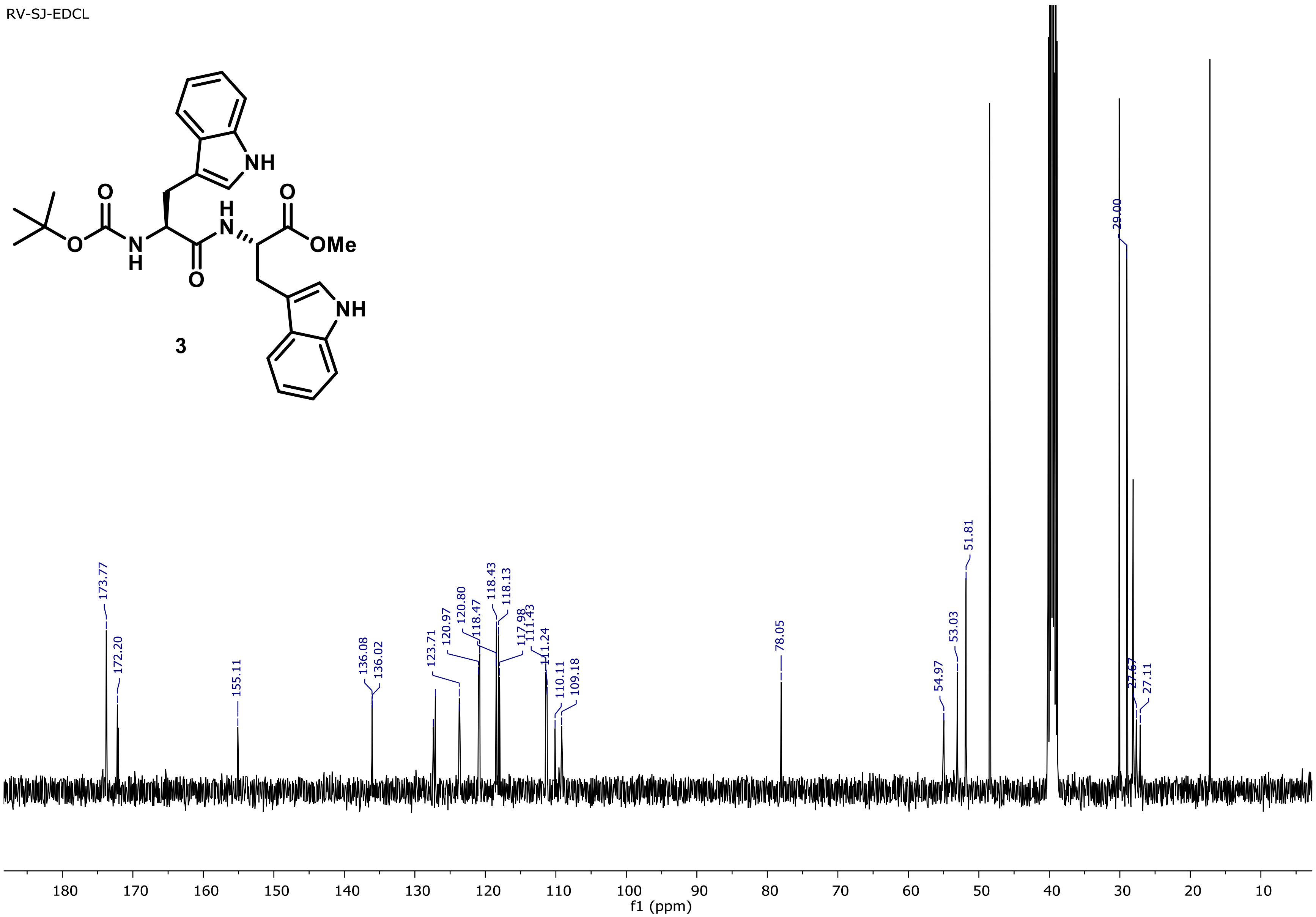
NH
O
H
N
O
OMe
N
H
O
O
NH
3
173.77
172.20
155.11
136.08
136.02
123.71
120.97
120.80
118.47
118.43
118.13
117.98
111.43
111.24
110.11
109.18
78.05
54.97
53.03
51.81
29.00
27.67
27.11
180
170
160
150
140
130
120
110
100
90
80
70
60
50
40
30
20
10
f1 (ppm)

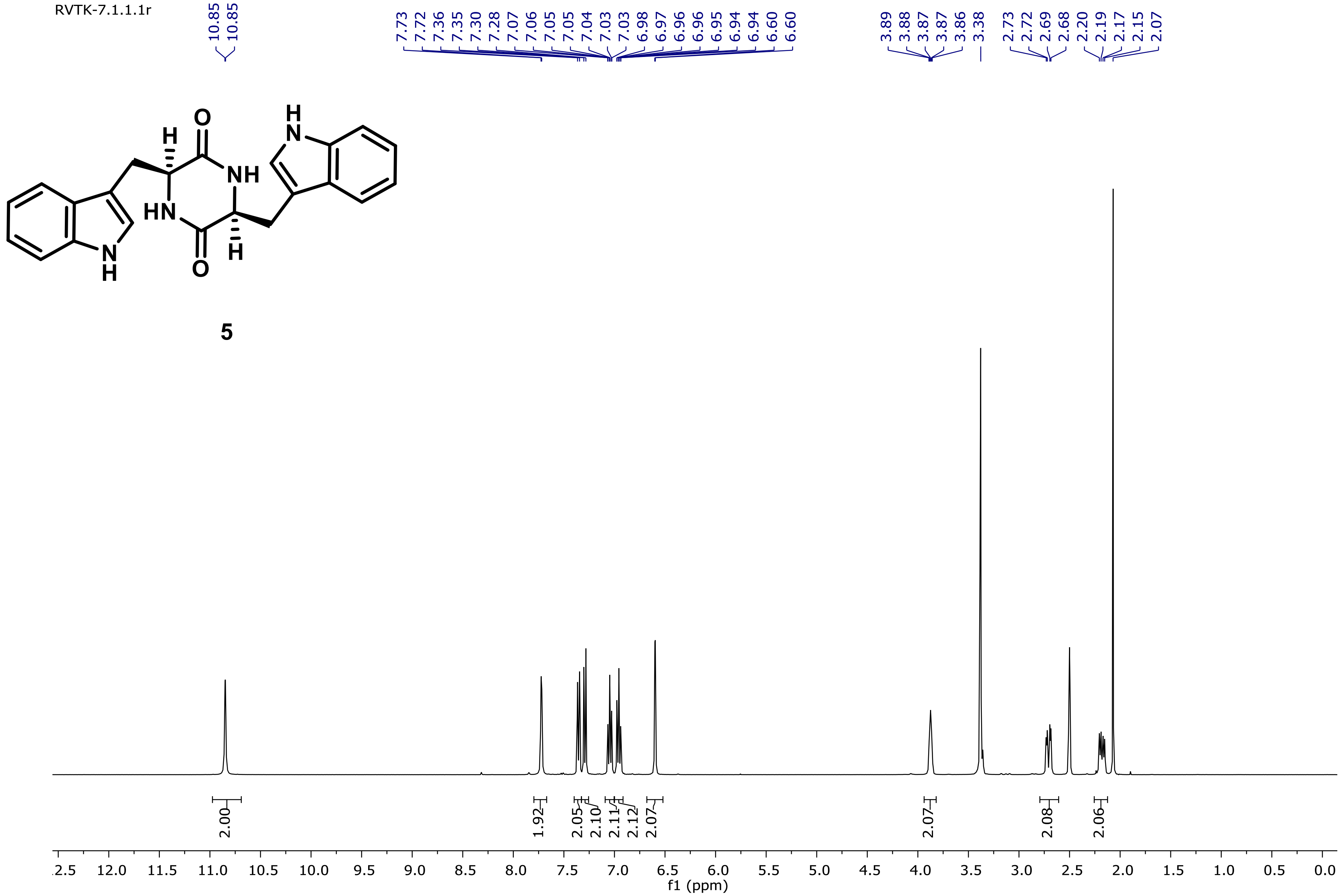
RVTK-7.1.1.1r
10.85
10.85
7.73
7.72
7.36
7.35
7.30
7.28
7.07
7.06
7.05
7.05
7.04
7.03
7.03
6.98
6.97
6.96
6.96
6.95
6.94
6.94
6.60
6.60
3.89
3.88
3.87
3.87
3.86
3.38
2.73
2.72
2.69
2.68
2.20
2.19
2.17
2.15
2.07
H
O
N
H
NH
HN
N
H
O
H
5
2.00
1.92
2.05
2.10
2.11
2.12
2.07
2.07
2.08
2.06
12.5
12.0
11.5
11.0
10.5
10.0
9.5
9.0
8.5
8.0
7.5
7.0
6.5
6.0
5.5
5.0
4.5
4.0
3.5
3.0
2.5
2.0
1.5
1.0
0.5
0.0
f1 (ppm)

RVTK-7.3.1.1r

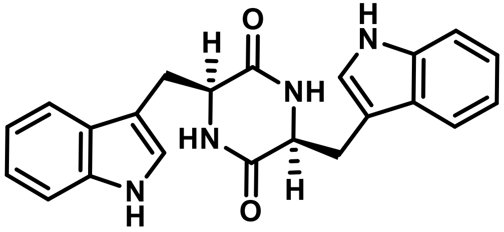

5

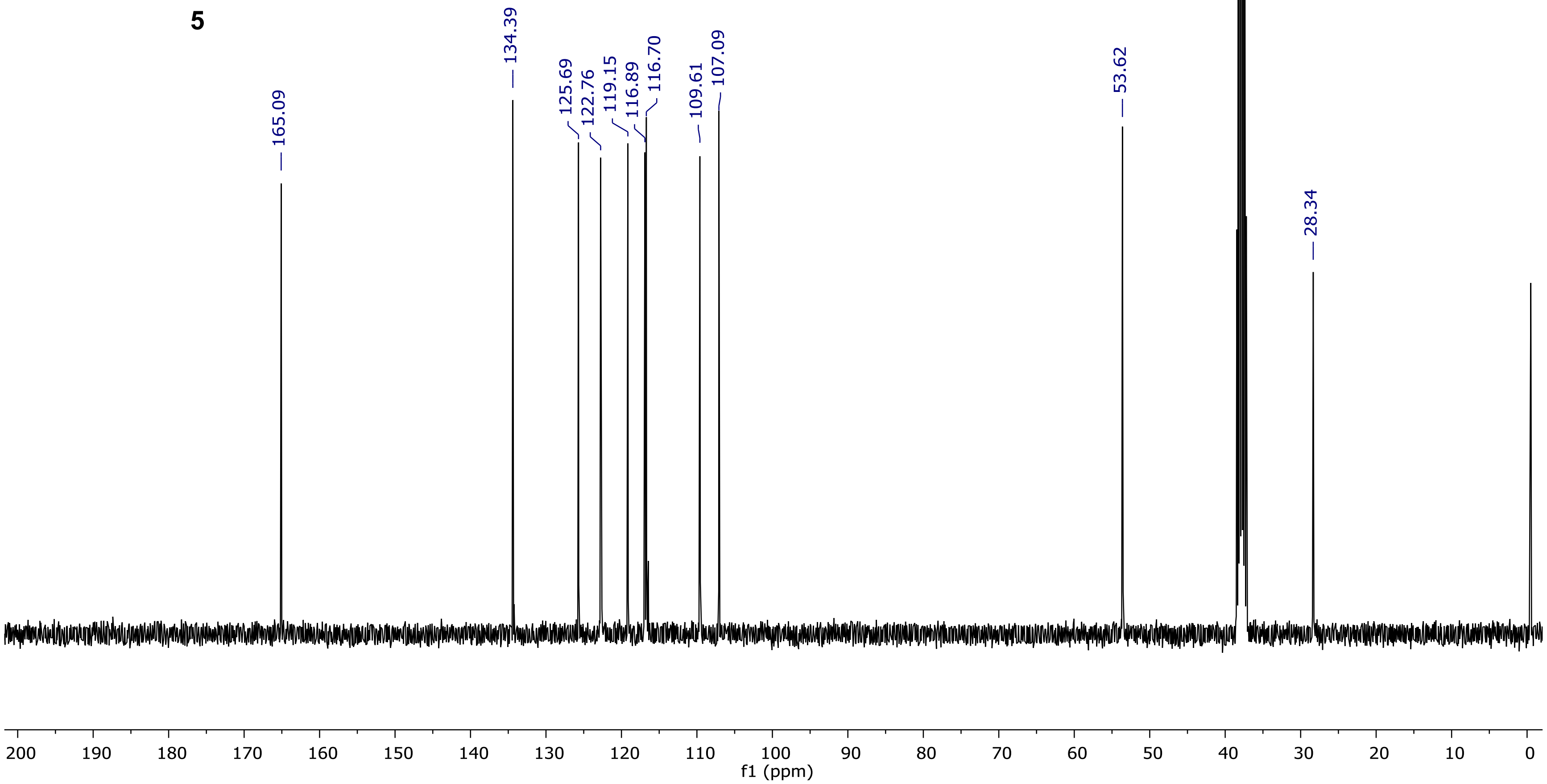
165.09
134.39
125.69
122.76
119.15
116.89
116.70
109.61
107.09
53.62
28.34
200 190 180 170 160 150 140 130 120 110 100 90 80 70 60 50 40 30 20 10 0
f1 (ppm)

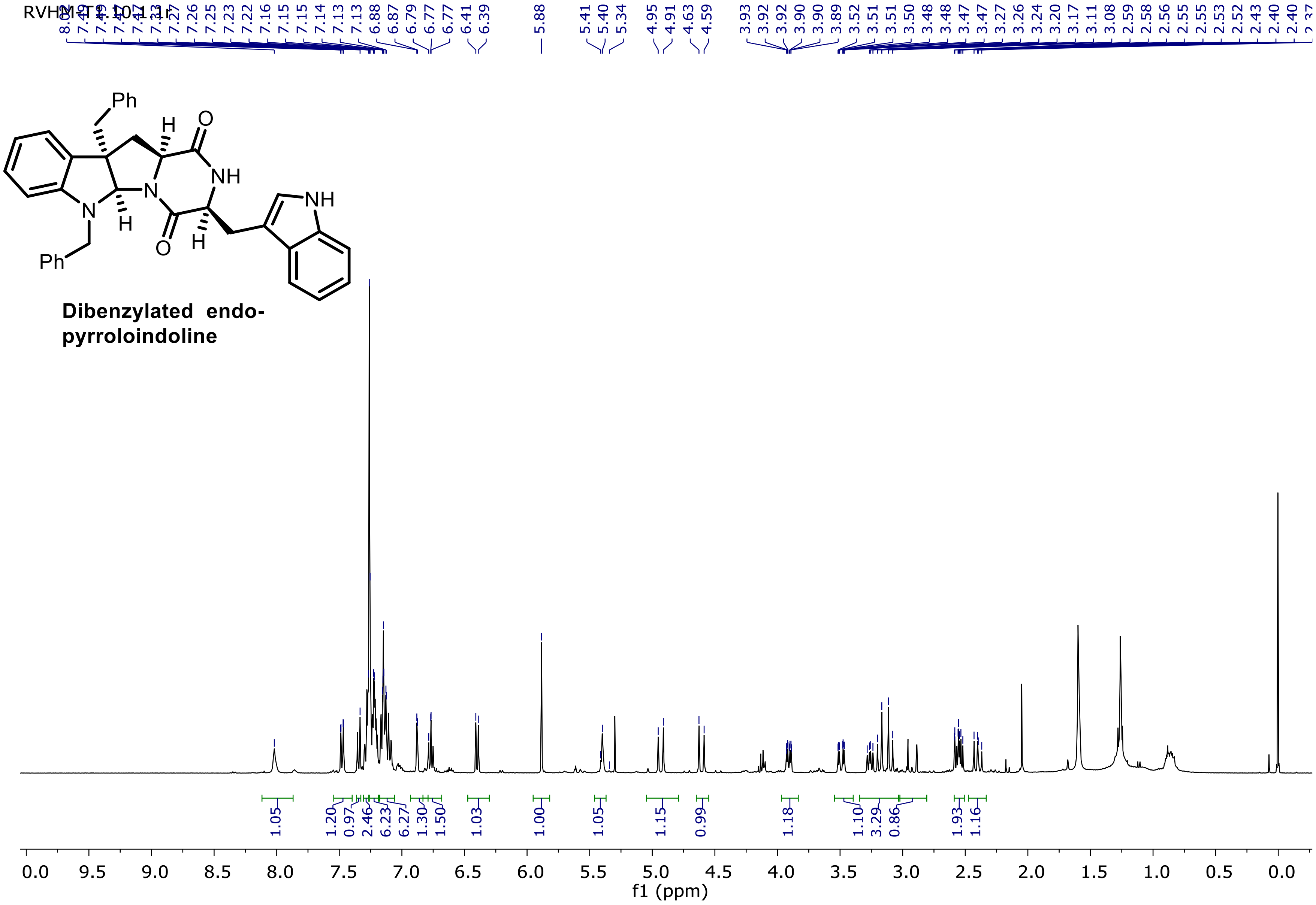

Dibenzylated endo-pyrroloindoline

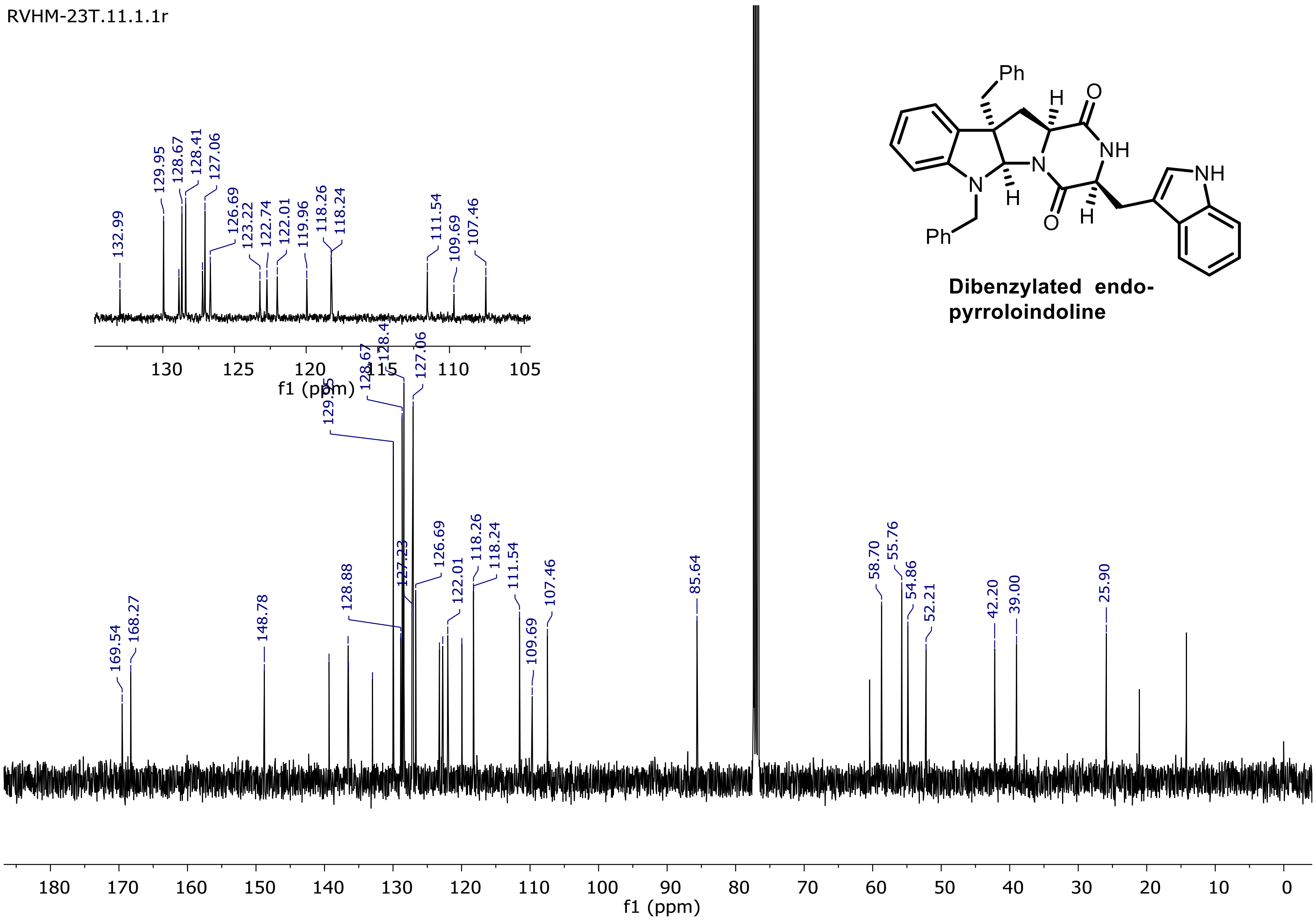

Dibenzylated endo-pyrroloindoline

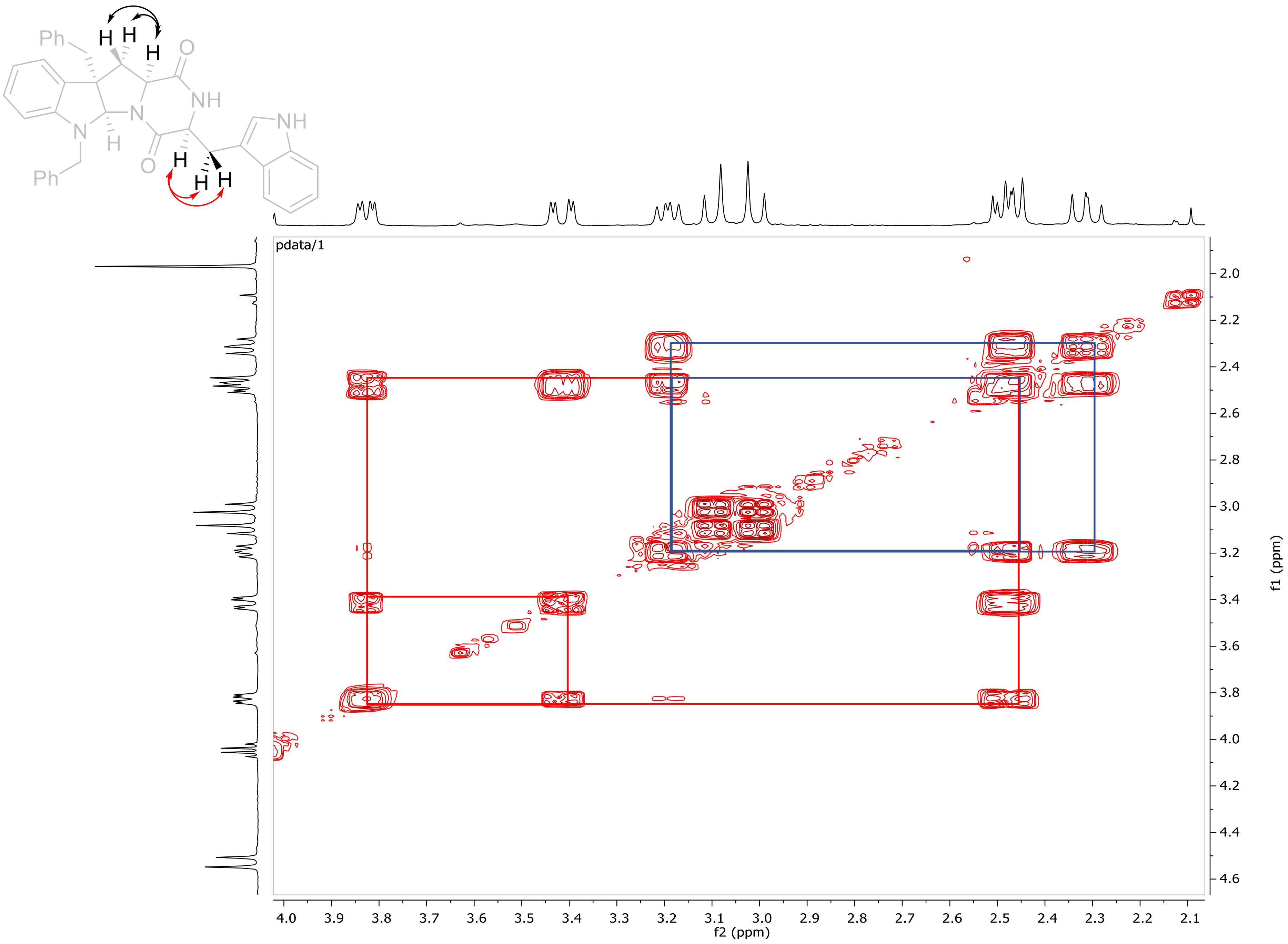

2D-COSY Spectra

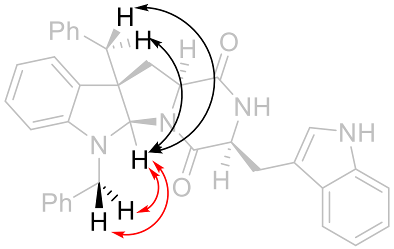

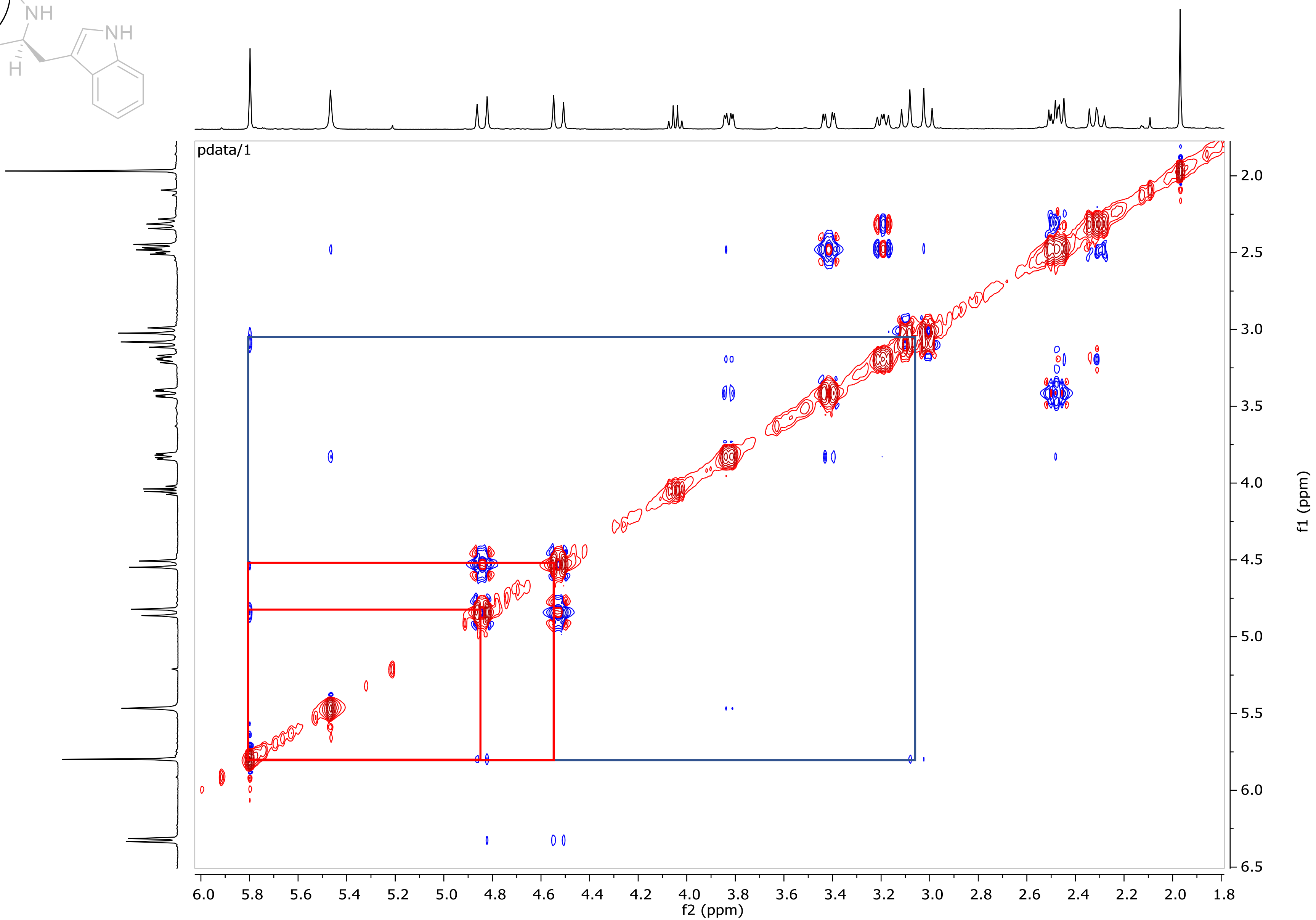

2D-NOESY Spectra

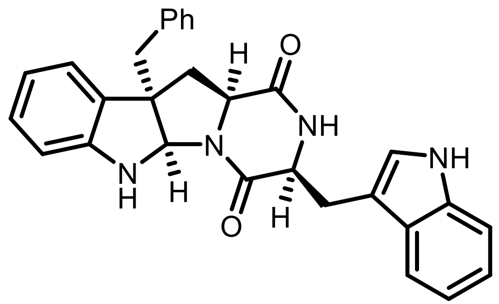

mono-benzylated endo-pyrroloindoline

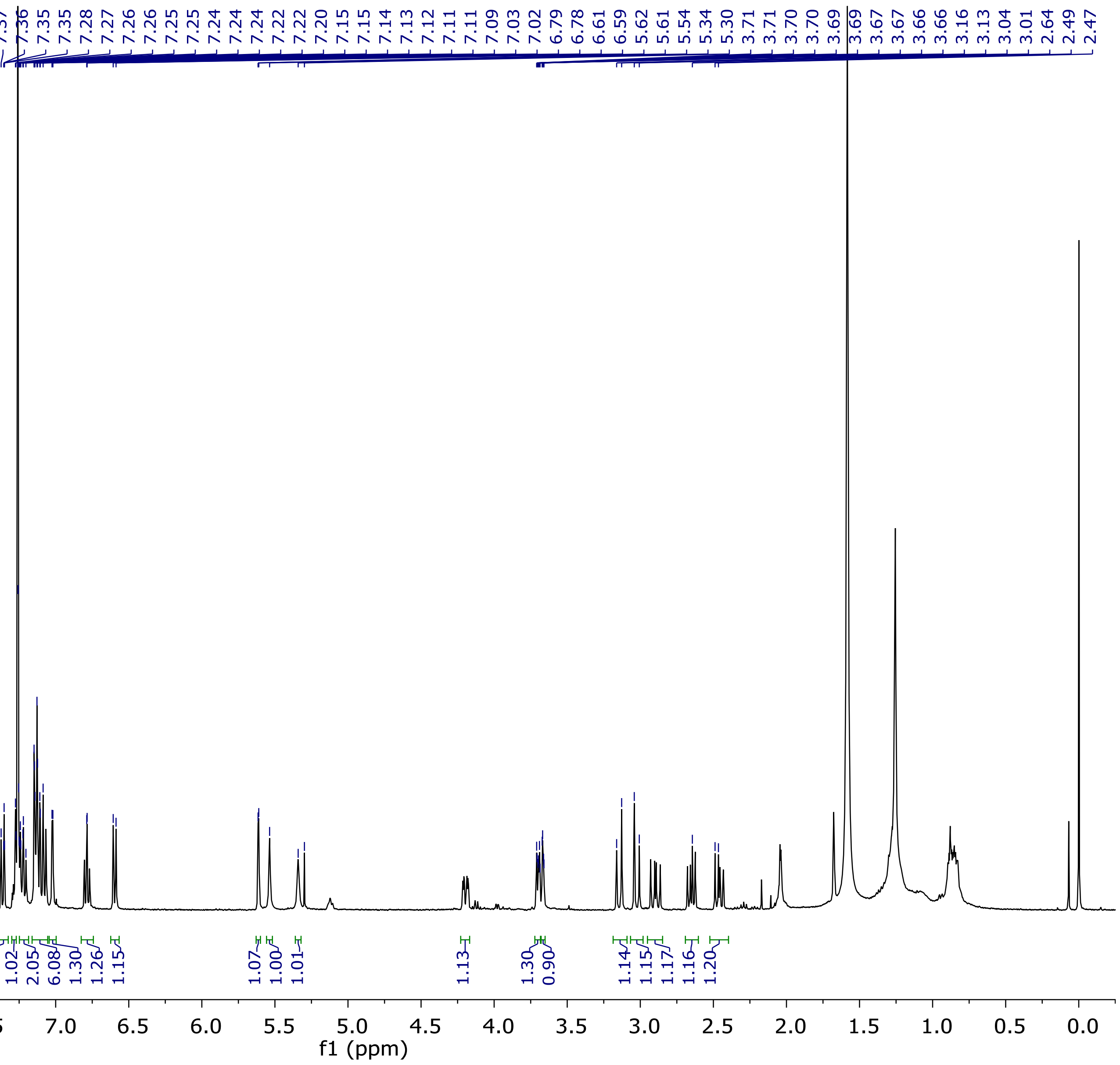

RVHM-23M.11.1.1r

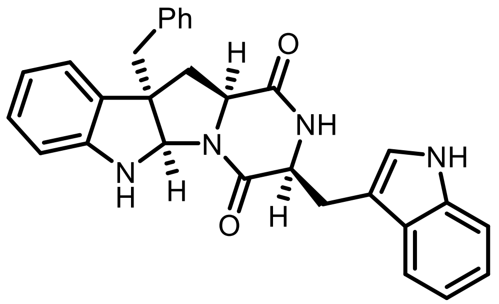

mono-benzylated endo-pyrroloindoline

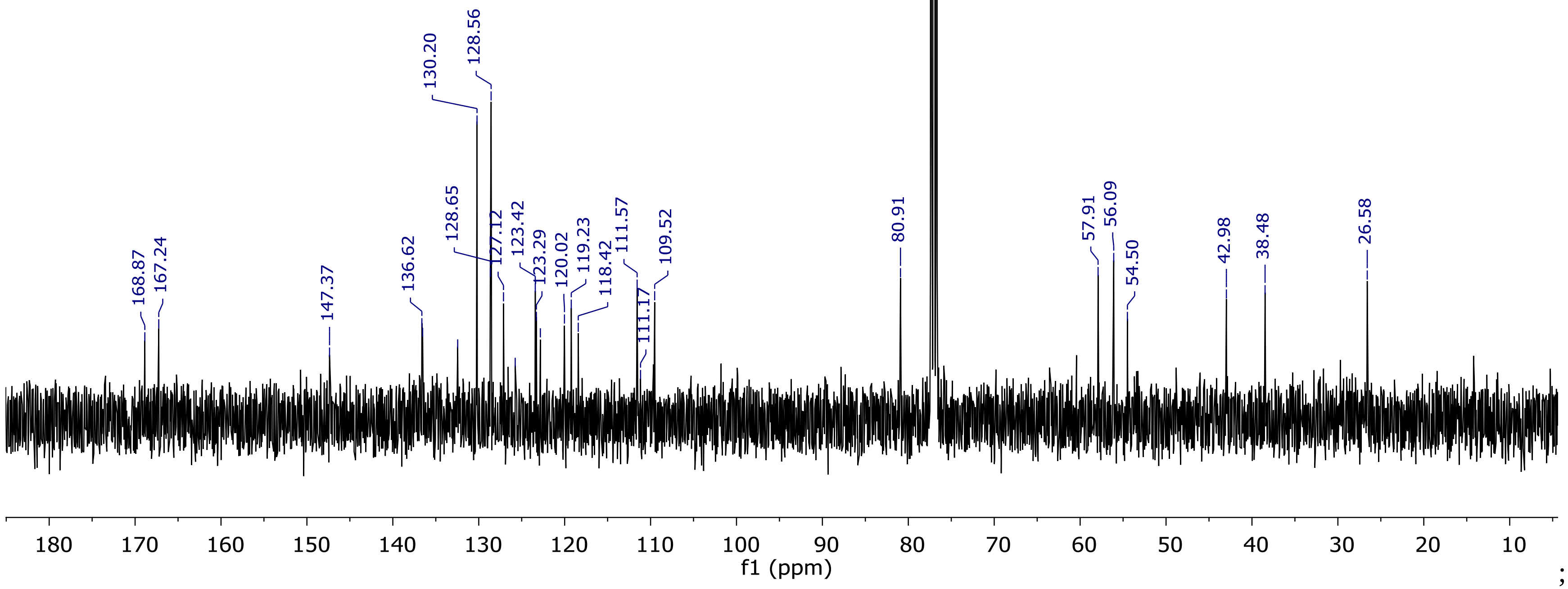

;.

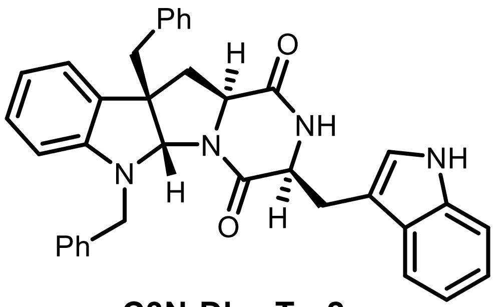

C3N-Dbn-Trp2

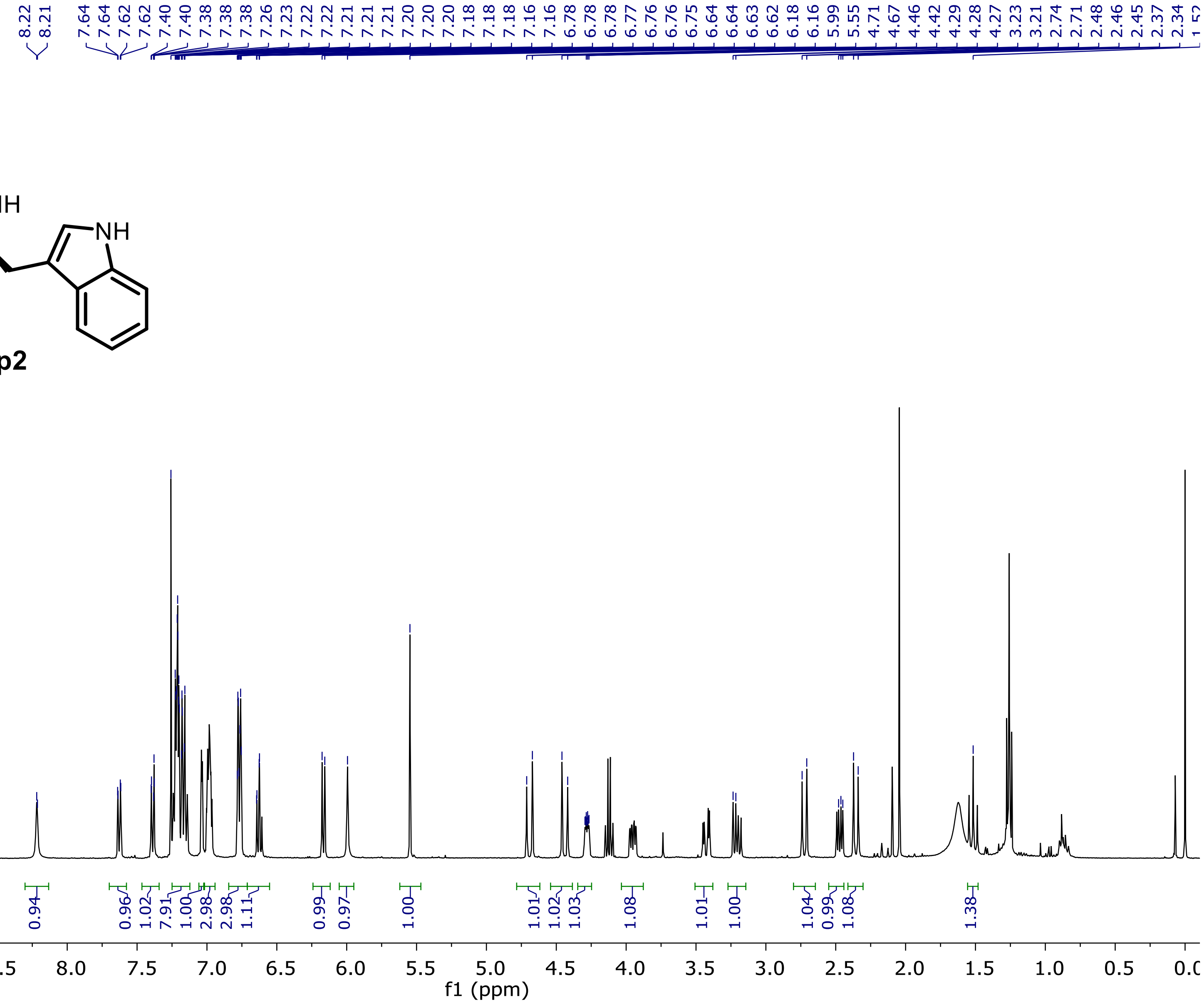
RVHM-20BT.10.1.1r
8.22
8.21
7.64
7.64
7.62
7.62
7.40
7.40
7.38
7.38
7.38
7.26
7.23
7.22
7.22
7.21
7.21
7.21
7.20
7.20
7.20
7.18
7.18
7.18
7.16
7.16
6.78
6.78
6.77
6.76
6.76
6.75
6.64
6.64
6.63
6.62
6.18
6.16
5.99
5.55
4.71
4.67
4.46
4.42
4.29
4.28
4.27
3.23
3.21
2.74
2.71
2.48
2.46
2.45
2.37
2.34
0.94
0.96
1.02
7.91
1.00
2.98
2.98
1.11
0.99
0.97
1.00
1.01
1.02
1.03
1.08
1.01
1.00
1.04
0.99
1.08
1.38
10.0
9.5
9.0
8.5
8.0
7.5
7.0
6.5
6.0
5.5
5.0
4.5
4.0
3.5
3.0
2.5
2.0
1.5
1.0
0.5
f1 (ppm)

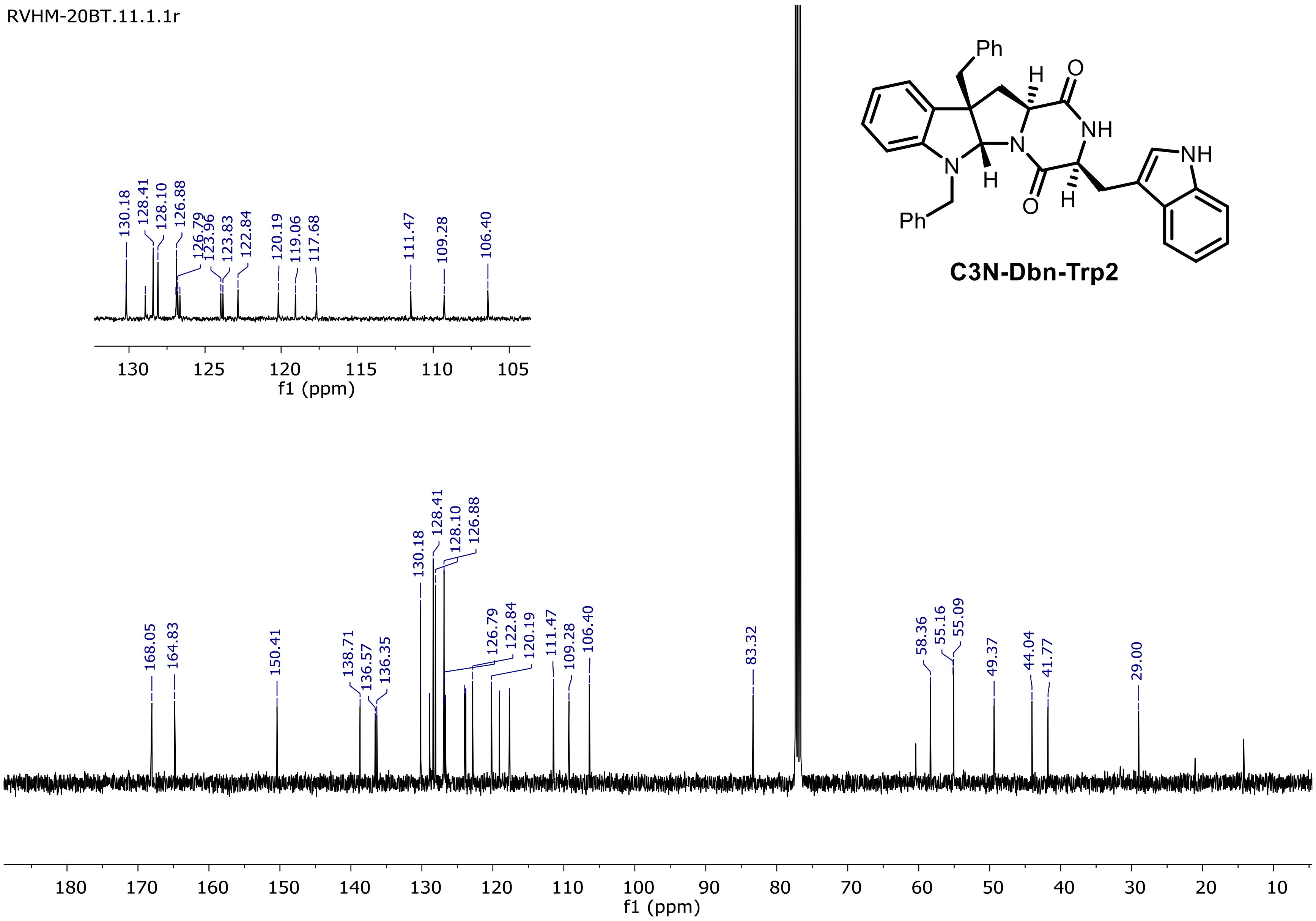
RVHM-20BT.11.1.1r
130.18
128.41
128.10
126.88
126.79
123.96
123.83
122.84
120.19
119.06
117.68
111.47
109.28
106.40
130 125 120 115 110 105
f1 (ppm)
Ph
H
O
NH
NH
N
N
H
H
O
Ph
C3N-Dbn-Trp2
168.05
164.83
150.41
138.71
136.57
136.35
130.18
128.41
128.10
126.88
126.79
122.84
120.19
111.47
109.28
106.40
83.32
58.36
55.16
55.09
49.37
44.04
41.77
29.00
180 170 160 150 140 130 120 110 100 90 80 70 60 50 40 30 20 10
f1 (ppm)

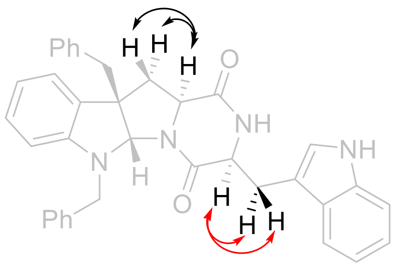

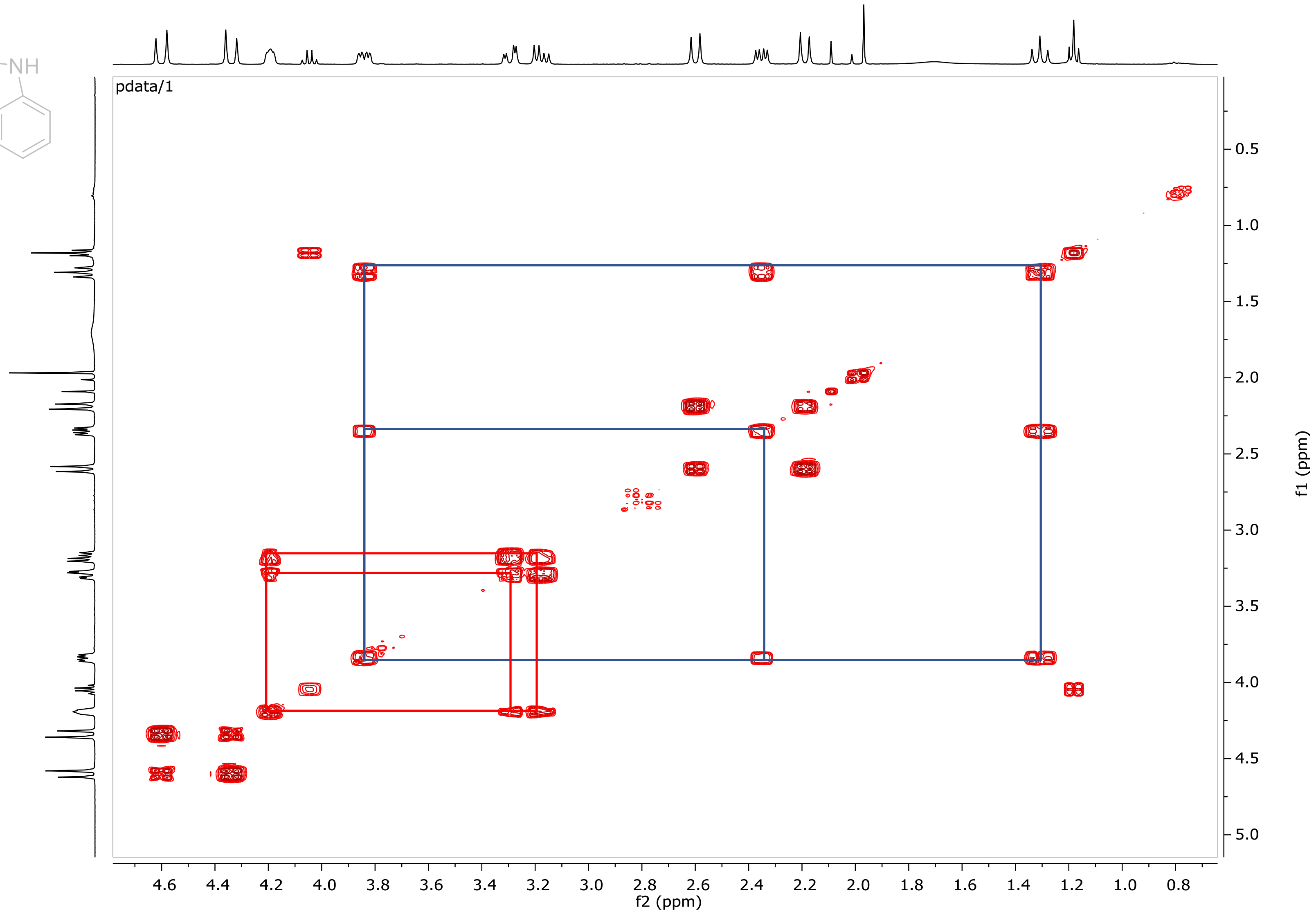

2D-COSY Spectra

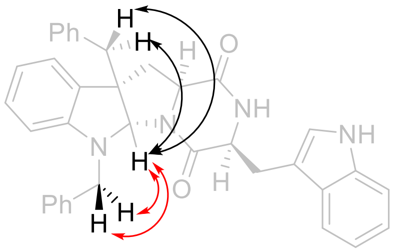

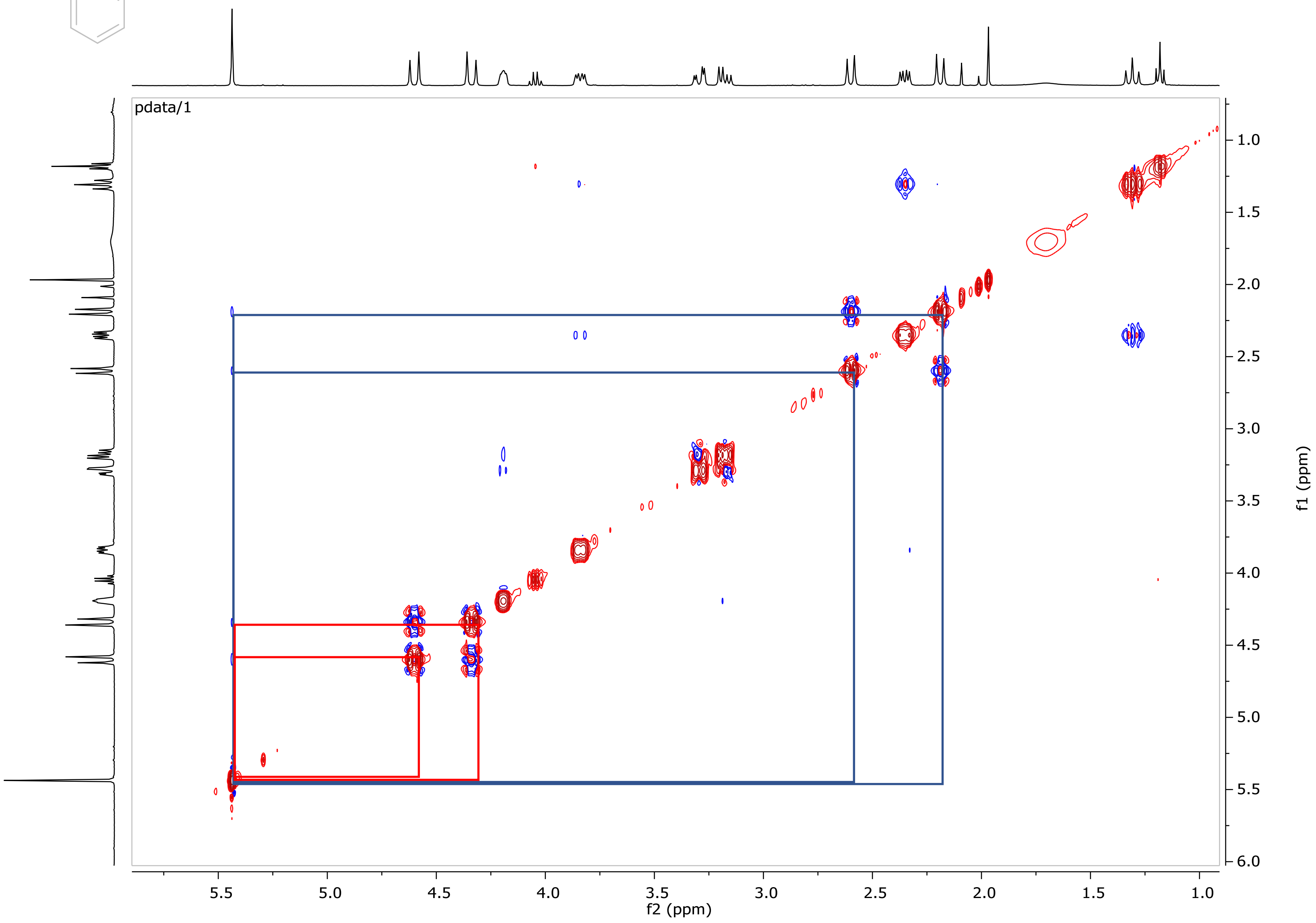

2D-NOESY Spectra

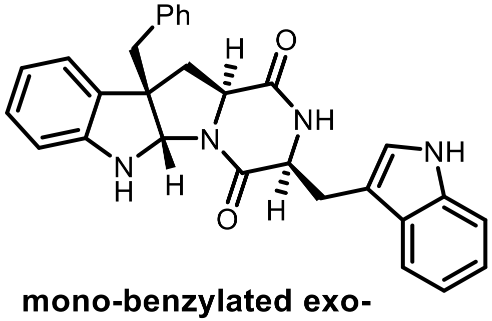

mono-benzylated exo-pyrroloindoline

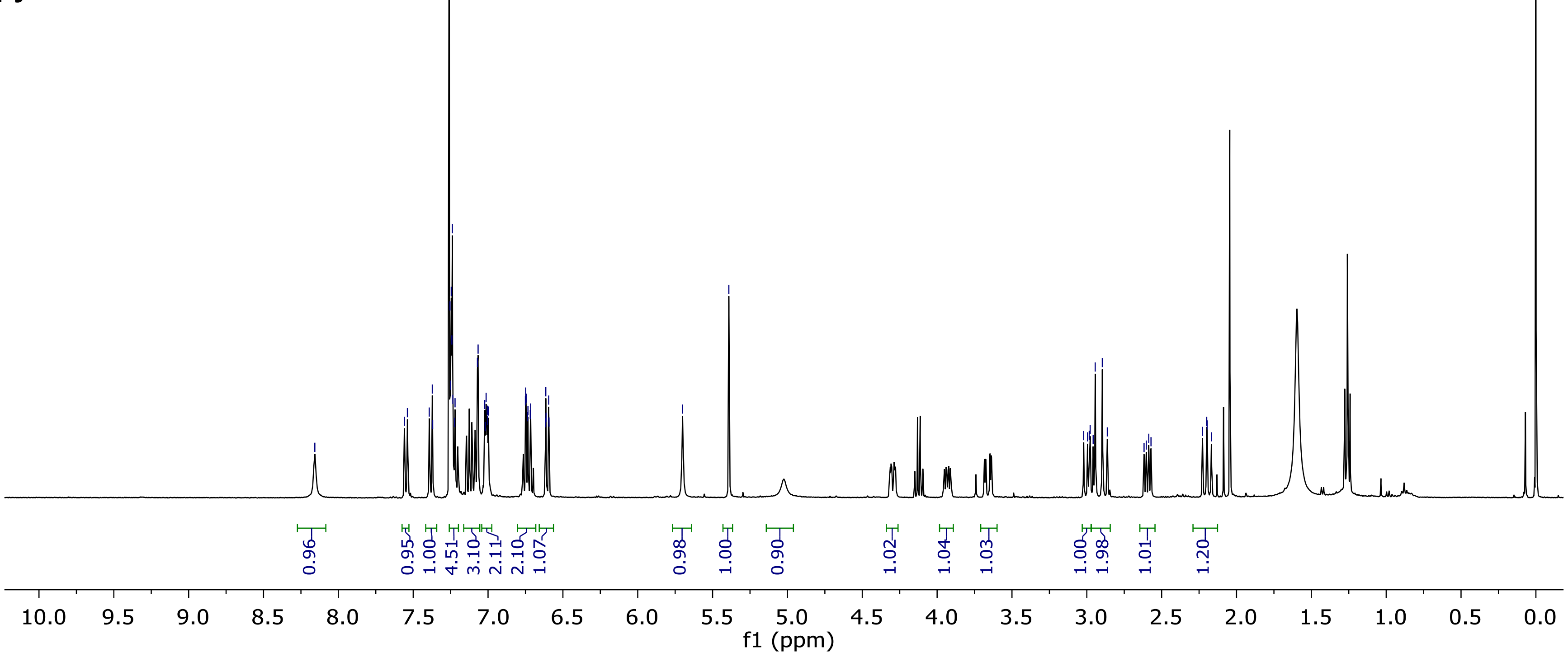

RVHM-20BB.11.1.1r

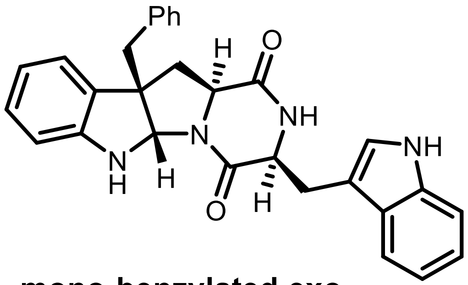

mono-benzylated exo-pyrroloindoline

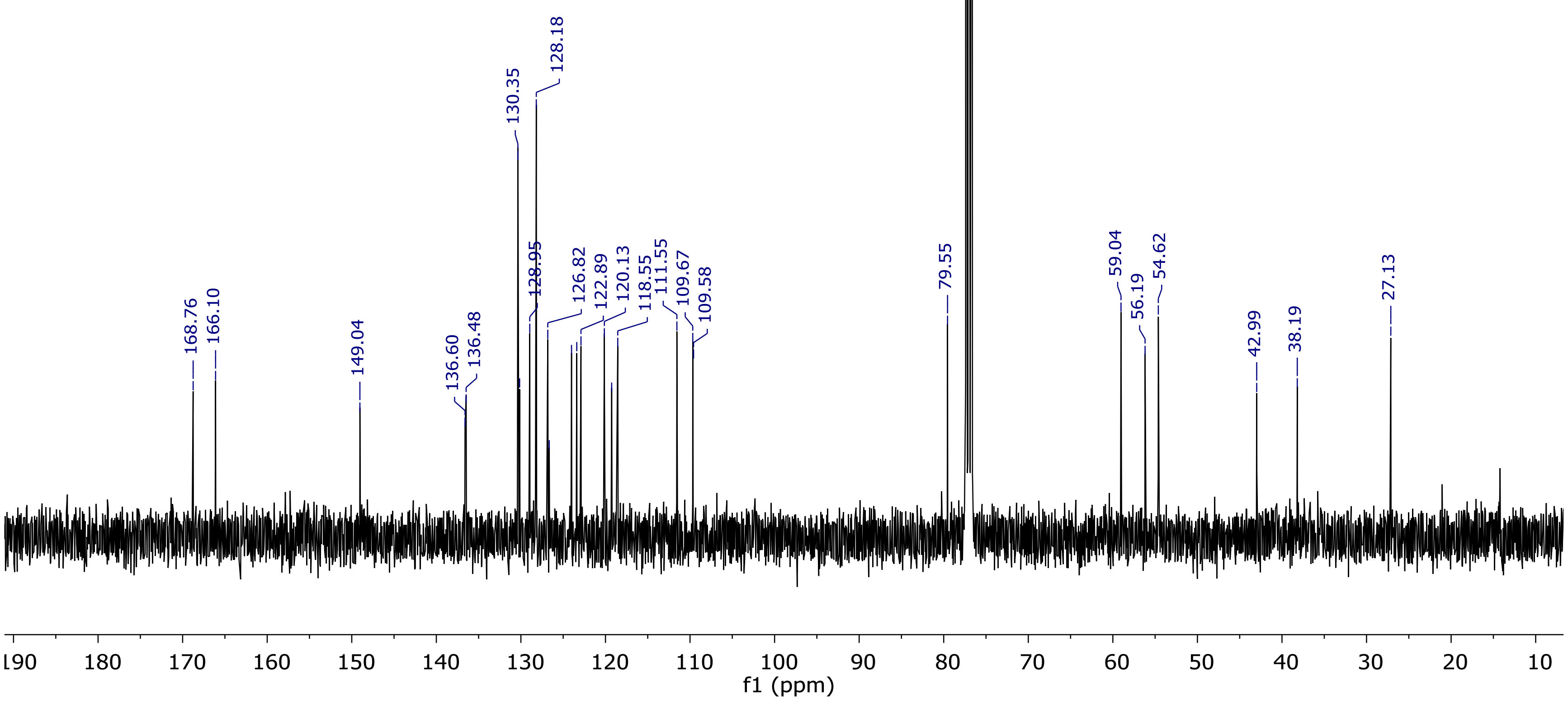

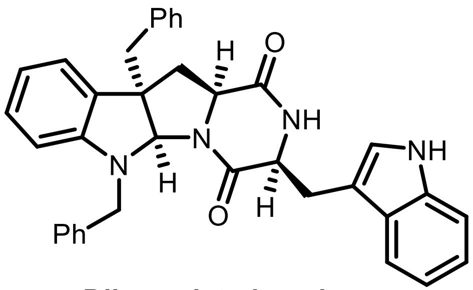

Dibenzylated endo-pyrroloindoline

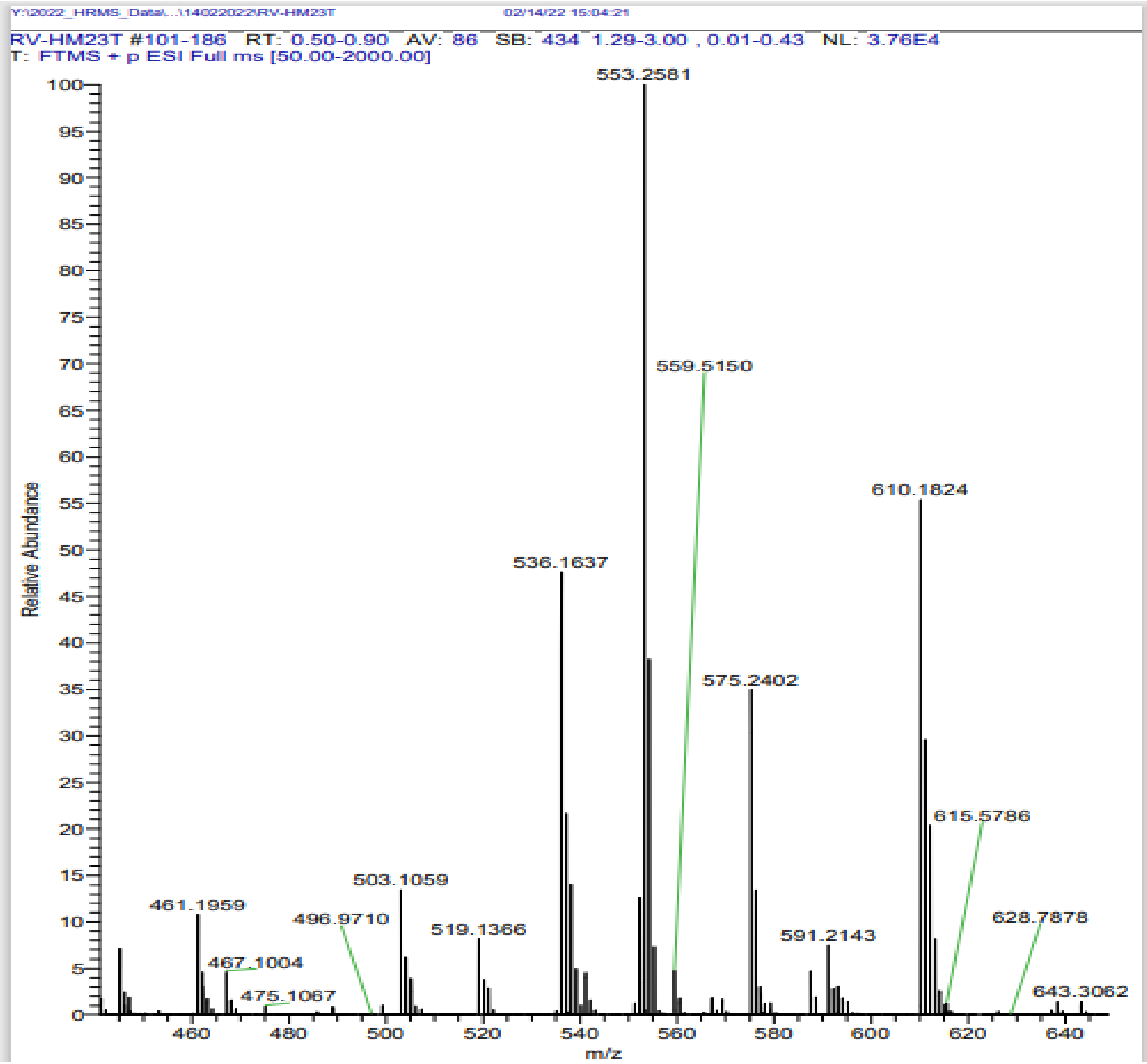

(M+H)$^{+}$:

Clcd:553.2589

Found:553.2581

1.4459ppm

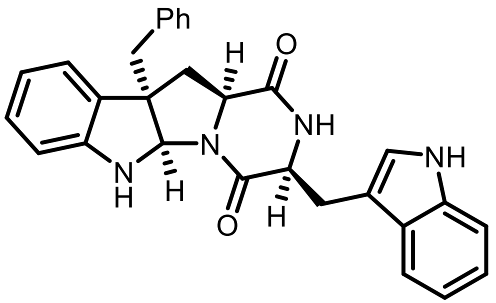

mono-benzylated endo-pyrroloindoline

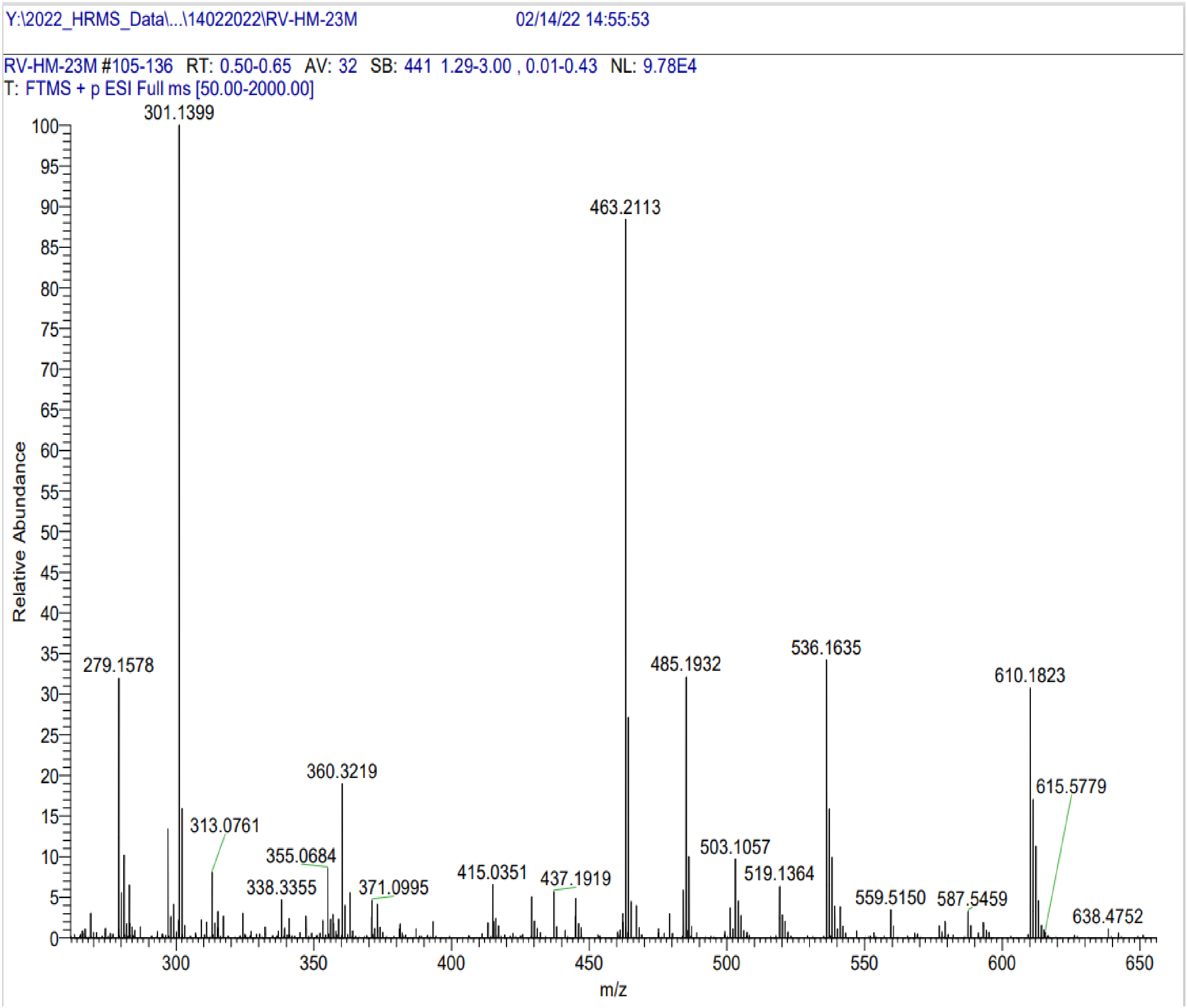

(M+H)$^{+}$:

Calcd:463.2129

Found:463.2113

3.4541 ppm

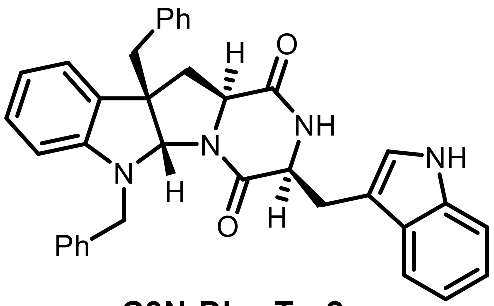

C3N-Dbn-Trp2

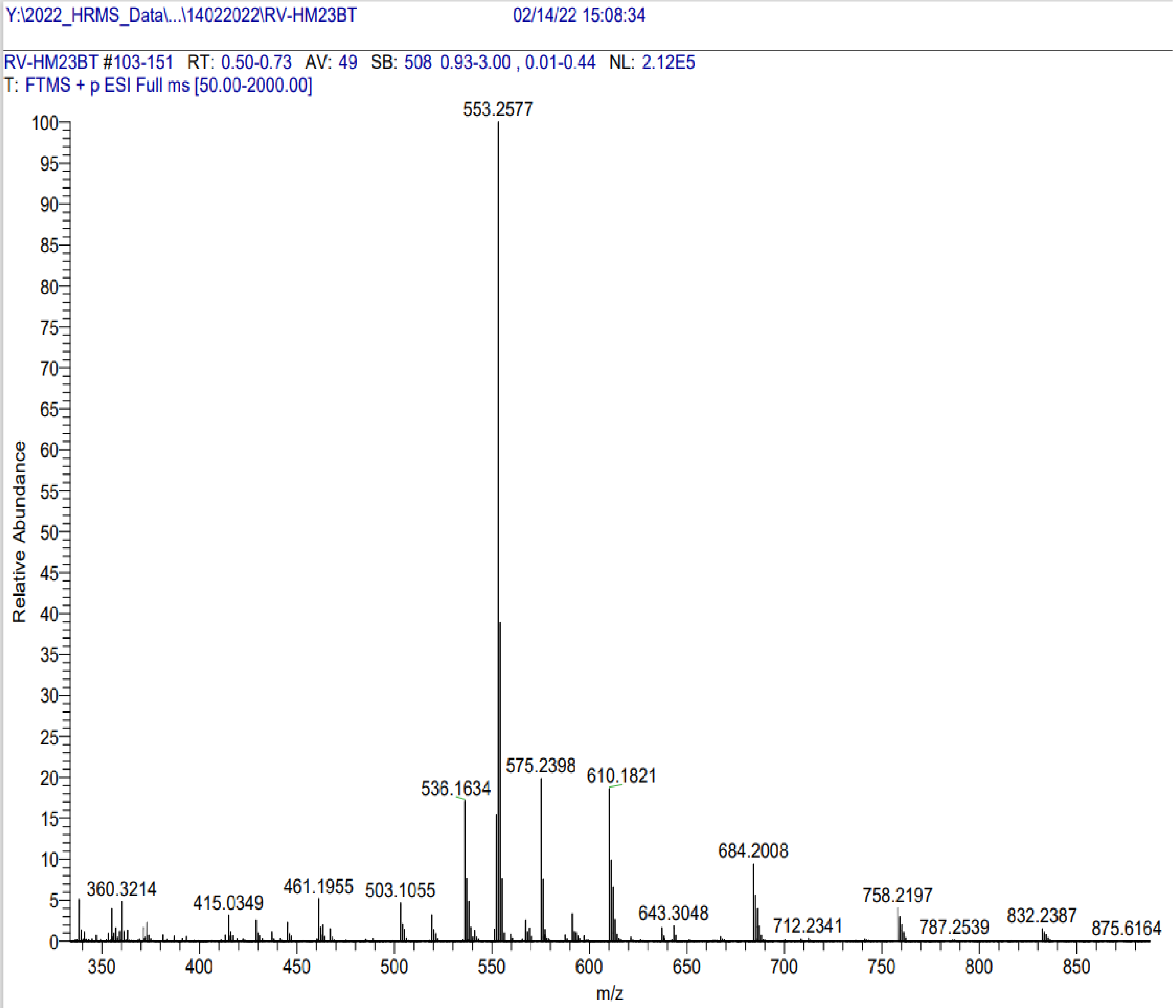

(M+H) [+]:

Cacld:553.2589

found:553.2577

2.1689 ppm

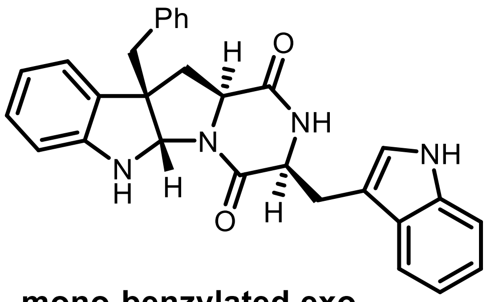

mono-benzylated exo-pyrroloindoline

(M+H)$^{+}$:

Cacld:463.2129

Found:463.2116

2.8064 ppm

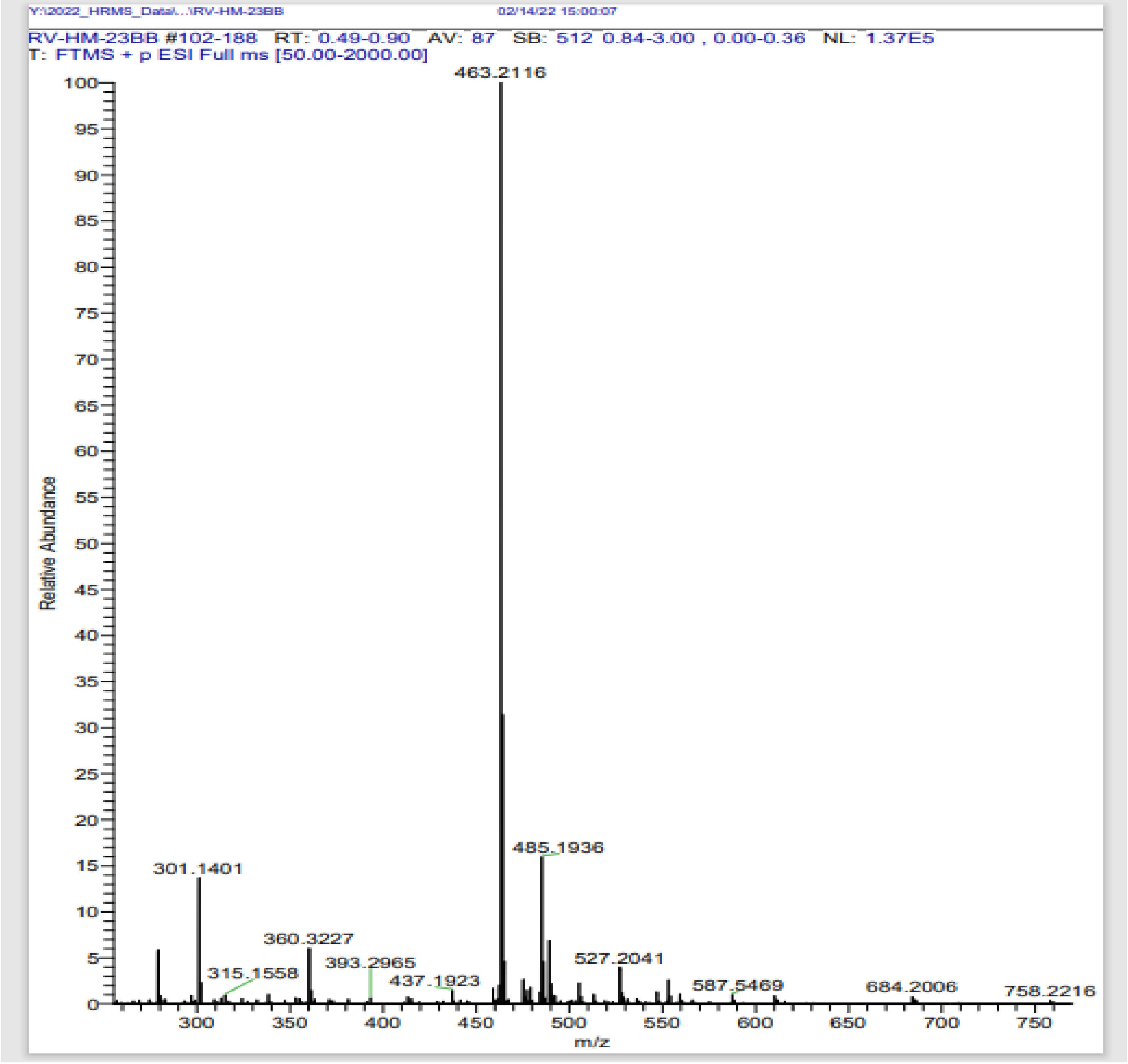

Supplement: Supplementary file 2 — Supplementary Information [file 42004_2024_1225_MOESM2_ESM.pdf]
