# Supplementary material for: Exploring bat-inspired cyclic tryptophan diketopiperazines as ABCB1 Inhibitors
Source: Commun Chem. 2024 Jul 13;7:158. doi: 10.1038/s42004-024-01225-z (PMC11246513; doi:10.1038/s42004-024-01225-z)
Supplement: Supplementary file 3 — Description of Additional Supplementary Files [file 42004_2024_1225_MOESM3_ESM.pdf]

Description of Additional Supplementary Files

File name: Supplementary Video 1

Description: Molecular interactions of the compounds with ABCB1. Left: Cyclo-(L-Trp-L-Trp) (Ligand 1: orange, ligand 2 : magenta) Middle: Cyclo-(L-1-methyl-Trp-L1-methyl-Trp) (Ligand 1: orange, ligand 2: magenta) Right: C3N-Dbn-Trp2 (Ligand 1: orange, ligand 2: magenta)
