# Supplementary material for: Exploring bat-inspired cyclic tryptophan diketopiperazines as ABCB1 Inhibitors
Source: Commun Chem. 2024 Jul 13;7:158. doi: 10.1038/s42004-024-01225-z (PMC11246513; doi:10.1038/s42004-024-01225-z)
Supplement: Supplementary file 5 — Reporting Summary [file 42004_2024_1225_MOESM5_ESM.pdf]

nature portfolio

Corresponding author(s): Koji Itahana, Peter J. Bond, Rajesh Viswanathan

Last updated by author(s): Jun 11, 2024

Reporting Summary

Nature Portfolio wishes to improve the reproducibility of the work that we publish. This form provides structure for consistency and transparency in reporting. For further information on Nature Portfolio policies, see our Editorial Policies and the Editorial Policy Checklist.

Statistics

For all statistical analyses, confirm that the following items are present in the figure legend, table legend, main text, or Methods section.

n/a	Confirmed	
☐	☒	The exact sample size (*n*) for each experimental group/condition, given as a discrete number and unit of measurement
☐	☒	A statement on whether measurements were taken from distinct samples or whether the same sample was measured repeatedly
☐	☒	The statistical test(s) used AND whether they are one- or two-sided<br>*Only common tests should be described solely by name; describe more complex techniques in the Methods section.*
☒	☐	A description of all covariates tested
☒	☐	A description of any assumptions or corrections, such as tests of normality and adjustment for multiple comparisons
☐	☒	A full description of the statistical parameters including central tendency (e.g. means) or other basic estimates (e.g. regression coefficient) AND variation (e.g. standard deviation) or associated estimates of uncertainty (e.g. confidence intervals)
☒	☐	For null hypothesis testing, the test statistic (e.g. *F*, *t*, *r*) with confidence intervals, effect sizes, degrees of freedom and *P* value noted<br>*Give P values as exact values whenever suitable.*
☒	☐	For Bayesian analysis, information on the choice of priors and Markov chain Monte Carlo settings
☒	☐	For hierarchical and complex designs, identification of the appropriate level for tests and full reporting of outcomes
☒	☐	Estimates of effect sizes (e.g. Cohen's *d*, Pearson's *r*), indicating how they were calculated

Our web collection on statistics for biologists contains articles on many of the points above.

Software and code

Policy information about availability of computer code

Data collection	AutoDock Vina and GROMACS (version 2018)
Data analysis	AutoDock Vina, GROMACS (version 2018) and g_mmpbsa

For manuscripts utilizing custom algorithms or software that are central to the research but not yet described in published literature, software must be made available to editors and reviewers. We strongly encourage code deposition in a community repository (e.g. GitHub). See the Nature Portfolio guidelines for submitting code & software for further information.

Data

Policy information about availability of data

All manuscripts must include a data availability statement. This statement should provide the following information, where applicable:

- Accession codes, unique identifiers, or web links for publicly available datasets
- A description of any restrictions on data availability
- For clinical datasets or third party data, please ensure that the statement adheres to our policy

We include a data availability statement in the manuscript.

Human research participants

Policy information about studies involving human research participants and Sex and Gender in Research.

Reporting on sex and gender	We don't have the results from samples taken from human research participants.
Population characteristics	We don't have the results from samples taken from human research participants.
Recruitment	We don't have the results from samples taken from human research participants.
Ethics oversight	We don't have the results from samples taken from human research participants.

Note that full information on the approval of the study protocol must also be provided in the manuscript.

Field-specific reporting

Please select the one below that is the best fit for your research. If you are not sure, read the appropriate sections before making your selection.

☒ Life sciences ☐ Behavioural & social sciences ☐ Ecological, evolutionary & environmental sciences

For a reference copy of the document with all sections, see nature.com/documents/nr-reporting-summary-flat.pdf

Life sciences study design

All studies must disclose on these points even when the disclosure is negative.

Sample size	The number of samples for each figure is at least three and is given for each experiment in the figure legend.
Data exclusions	No data were excluded from any of the analyses in this study.
Replication	All experiments were performed independently at least three times, unless otherwise stated in the figure legends. Data from each experiment were reproducible with minimal standard deviations.
Randomization	No randomization techniques were used.
Blinding	No blinding was used.

Reporting for specific materials, systems and methods

We require information from authors about some types of materials, experimental systems and methods used in many studies. Here, indicate whether each material, system or method listed is relevant to your study. If you are not sure if a list item applies to your research, read the appropriate section before selecting a response.

Materials & experimental systems

n/a	Involved in the study	
☐	☒	Antibodies
☐	☒	Eukaryotic cell lines
☒	☐	Palaeontology and archaeology
☒	☐	Animals and other organisms
☒	☐	Clinical data
☒	☐	Dual use research of concern

Methods

n/a	Involved in the study	
☒	☐	ChIP-seq
☐	☒	Flow cytometry
☒	☐	MRI-based neuroimaging

Antibodies

Antibodies used	H2AX (A303-837A, Bethyl Laboratories, Inc.), γH2AX (05-636, Millipore), tubulin (ab44928, Abcam), actin (MAB1501, Millipore), ABCB1 (GTX23364, GeneTex; sc-55510, Santa Cruz), ABCC1 (67228-1-Ig, Proteintech), ABCG2 (sc-58222, Santa Cruz), horseradish peroxidase (HRP)-conjugated secondary antibody (Jackson Immuno Research laboratories), and fluorescence-labeled secondary antibodies (Thermo Scientific Scientific).
Validation	All antibodies used in this study were validated by their respective suppliers.

Eukaryotic cell lines

Policy information about cell lines and Sex and Gender in Research

Cell line source(s)	PaKiT03 cells were derived from P. alecto kidney and immortalized with large T-antigen. HCT-15, HEK293T, and BeWo cells were purchased from American Type Culture Collection (ATCC).
Authentication	All commercial cell lines were authenticated by the suppliers. Primary bat cell lines were not authenticated.
Mycoplasma contamination	All cell lines used in this study were tested negative for mycoplasma.
Commonly misidentified lines (See ICLAC register)	None.

Flow Cytometry

Plots

Confirm that:

☒ The axis labels state the marker and fluorochrome used (e.g. CD4-FITC).

☒ The axis scales are clearly visible. Include numbers along axes only for bottom left plot of group (a 'group' is an analysis of identical markers).

☒ All plots are contour plots with outliers or pseudocolor plots.

☒ A numerical value for number of cells or percentage (with statistics) is provided.

Methodology

Sample preparation	Cells were pre-treated with DMSO (a negative control) or the compounds and then co-incubated with the fluorescent substrate. The stained cells were trypsinized, washed once with 1x PBS, and suspended in 1x PBS for flow cytometry.
Instrument	MACSQuant Analyzer 10 (Miltenyi Biotec)
Software	FlowJo software
Cell population abundance	CRISPR/Cas9-mediated knockouts were performed on the ABCB1, ABCC1, and ABCG2 genes. Electroporated cells were incubated with fluorescent substrate for each transporter. Cells were sorted based on fluorescence accumulation. Knockout efficiency was then analysed by Western blotting.
Gating strategy	No extensive gating strategy was used.

☐ Tick this box to confirm that a figure exemplifying the gating strategy is provided in the Supplementary Information.
